# *In vivo *rescue of alveolar macrophages from SP-A knockout mice with exogenous SP-A nearly restores a wild type intracellular proteome; actin involvement

**DOI:** 10.1186/1477-5956-9-67

**Published:** 2011-10-28

**Authors:** David S Phelps, Todd M Umstead, Omar A Quintero, Christopher M Yengo, Joanna Floros

**Affiliations:** 1 Center for Host defense, Inflammation, and Lung Disease (CHILD) Research and Department of Pediatrics, The Pennsylvania State University College of Medicine, Hershey, PA 17033, USA; 2 Department of Cellular and Molecular Physiology, The Pennsylvania State University College of Medicine, Hershey, PA 17033, USA; 3 Department of Obstetrics and Gynecology, The Pennsylvania State University College of Medicine, Hershey, PA 17033, USA

## Abstract

**Background:**

Mice lacking surfactant protein-A (SP-A-/-; knockout; KO) exhibit increased vulnerability to infection and injury. Although many bronchoalveolar lavage (BAL) protein differences between KO and wild-type (WT) are rapidly reversed in KO after infection, their clinical course is still compromised. We studied the impact of SP-A on the alveolar macrophage (AM) proteome under basal conditions. Male SP-A KO mice were SP-A-treated (5 micrograms/mouse) and sacrificed in 6 or 18 hr. The AM proteomes of KO, SP-A-treated KO, and WT mice were studied by 2D-DIGE coupled with MALDI-ToF/ToF and AM actin distribution was examined by phalloidon staining.

**Results:**

We observed: a) significant differences from KO in WT or exogenous SP-A-treated in 45 of 76 identified proteins (both increases and decreases). These included actin-related/cytoskeletal proteins (involved in motility, phagocytosis, endocytosis), proteins of intracellular signaling, cell differentiation/regulation, regulation of inflammation, protease/chaperone function, and proteins related to Nrf2-mediated oxidative stress response pathway; b) SP-A-induced changes causing the AM proteome of the KO to resemble that of WT; and c) that SP-A treatment altered cell size and F-actin distribution.

**Conclusions:**

These differences are likely to enhance AM function. The observations show for the first time that acute *in vivo *SP-A treatment of KO mice, under basal or unstimulated conditions, affects the expression of multiple AM proteins, alters F-actin distribution, and can restore much of the WT phenotype. We postulate that the SP-A-mediated expression profile of the AM places it in a state of "readiness" to successfully conduct its innate immune functions and ensure lung health.

## Introduction

SP-A, a multi-functional protein, is known to play an important role in host defense. SP-A is a collectin, or collagenous lectin, that can recognize pathogen-associated molecular patterns (PAMP). The recognition and binding of PAMP is complex and may involve binding sites in addition to the C-type carbohydrate recognition domain. Although the direct interaction with pathogens constitutes one aspect of its host defense function, SP-A also plays a role in the clearance of particulate matter, allergens, and debris from the alveolar surface [1-5]. SP-A appears to have a regulatory role on the alveolar macrophage by influencing the expression of a number of cytokines, including TNF-α, IL-1β, and others [6-16], and cell surface molecules, such as CD11b (CR3), TLR2 and TLR4, the mannose receptor, scavenger receptor A, and CD14 [17-21]. Moreover, SP-A can help regulate redox balance [22-26], enhance bacterial phagocytosis by alveolar macrophages [27-30], contribute to bacterial killing [31-33], affect the development of dendritic cells [34], and provide an interface between innate and adaptive immunity [35]. Despite this diverse array of functions, many gaps remain in our knowledge of how SP-A influences lung host defense and the cell types it affects, especially under basal or unstimulated conditions.

SP-A-/- (knockout; KO) mice exhibit increased vulnerability to infection and injury. This has been illustrated with mouse models of pneumonia with organisms including *Klebsiella pneumoniae*, *Streptococcus pneumoniae*, *Pseudomonas aeruginosa*, *Pneumocystis carinii*, respiratory syncytial virus, and others [28,36-41]. Although the increased susceptibility was initially thought to be a consequence of the absence of the stimulatory effect of SP-A on phagocytosis, recent studies suggest a more complex picture. We have recently shown that in the absence of SP-A, baseline levels of many host defense molecules in bronchoalveolar lavage (BAL) samples [26,42] differ significantly (including both increases and decreases) from those in WT mice. However, although many of these differences in the SP-A KO mice are rapidly compensated for during infection and reach levels comparable to those of WT mice, the clinical course, and survival in particular [28] of the KO mice remains less favorable compared to that of the WT mice [27]. This may indicate that along with known direct effects of SP-A on phagocytosis and bacterial killing, there may be other direct and indirect effects of SP-A that may be instrumental in determining the clinical course and that these effects cannot occur in the absence of SP-A.

A likely source of these host defense deficits in the SP-A KO mouse is the alveolar macrophage, the primary effector cell for innate immunity in the lung. Although macrophages, which are derived from blood monocytes, are found throughout the body, their phenotype may vary depending on their environment. Alveolar macrophages exhibit a unique phenotype [43] that is clearly influenced by the presence of SP-A [17-21,27,28], but the extent of this influence is not entirely known. Moreover, virtually nothing is known about the *in vivo *effect of SP-A on the alveolar macrophage proteome under basal or unstimulated conditions, with regards to whether and which groups of proteins SP-A may affect under such conditions. However, based on recent *in vitro *studies, in response to LPS [44] or in extrapulmonary tissues [45], it appears that the integrity of the cytoskeleton is required in order for SP-A-mediated processes to occur.

In this study we tested the hypothesis that SP-A exerts a significant impact on the pattern of protein expression by the alveolar macrophage under basal or unstimulated conditions to make the alveolar macrophage ready and competent to subsequently mount a successful innate host defense response in response to various injurious agents. Towards this end we employed two-dimensional difference gel electrophoresis (2D-DIGE) to study *in vivo *the changes in the macrophage proteome in response to SP-A treatment of SP-A KO mice, the functional groups the changed proteins belong to, and whether the SP-A-induced changes render the proteome from alveolar macrophages of the KO mice more like that of wild type (WT) mice. SP-A KO mice treated with SP-A and sacrificed at different times post-treatment, as well as WT mice on the same C57BL/6 genetic background were used in this study. The data were subjected to various analytical methods to assess, in an unbiased fashion, the similarities and differences in the proteomes of our experimental groups, as well as, by considering the function of the changed proteins, to obtain insight into underlying mechanisms and the SP-A-mediated functions of the alveolar macrophage. Based on the findings a "proof of principle" experiment was carried out where changes were measured in the actin cytoskeleton.

## Results

### BAL cells

The cells from all BAL samples were subjected to total and differential cell counts to exclude any mice with underlying infectious or inflammatory processes. No evidence of inflammation or infection was seen in any of the mice. The total cell counts did not differ significantly from one another and all BAL samples consisted of > 95% macrophages (data not shown).

Protein content was the same in all samples (data not shown) and identical amounts of protein (20 μg) were loaded on all gels.

### 2-D DIGE results

#### Overview

**Figure 1:**
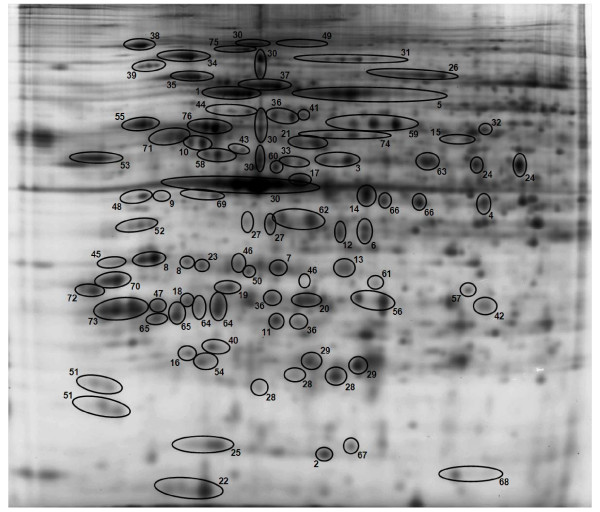
**Reference gel image of mouse alveolar macrophage proteins identified by 2D-DIGE**. Some of the proteins may include multiple spots reflecting different isoforms, fragments, or multimers (see also Additional File 3). 1, 65-kDa macrophage protein; 2, Actin related protein 2/3 complex, subunit 5; 3, Actin-related protein 3; 4, Actr2 protein; 5, Alpha-fetoprotein; 6, Annexin A2; 7, Annexin A4; 8, Anxa5 protein; 9, ArsA arsenite transporter, ATP-binding, homolog 1; 10, Atp5b protein; 11, Calpain, small subunit 1; 12, Capping protein (actin filament) muscle Z-line, alpha 2; 13, Capping protein (actin filament) muscle Z-line, beta isoform a; 14, Cathepsin D precursor; 15, Chaperonin subunit 2 (beta); 16, Chia protein; 17, Chitinase 3-like 3 precursor; 18, Chitinase-related protein MCRP; 19, Chloride intracellular channel 1; 20, Chloride intracellular channel 4; 21, CNDP dipeptidase 2; 22, Coactosin-like 1; 23, EF hand domain containing 2; 24, Eno1 protein (Alpha-enolase); 25, Eukaryotic translation initiation factor 5A; 26, Ezrin; 27, F-actin capping protein alpha-1 subunit; 28, Ferritin heavy chain 1; 29, Ferritin light chain 1; 30, Gamma-actin; 31, Gelsolin precursor; 32, Glucose-6-phosphate dehydrogenase X-linked; 33, Guanine deaminase; 34, Heat shock protein 1, beta; 35, Heat shock protein 5 precursor; 36, Heat shock protein 65; 37, Heat shock protein 8; 38, Heat shock protein 90, beta (Grp94), member 1; 39, Hematopoietic cell specific Lyn substrate 1; 40, Heme-binding protein; 41, Heterogeneous nuclear ribonucleoprotein K; 42, High mobility group 1 protein; 43, Hnrpf protein; 44, Kappa-B motif-binding phosphoprotein; 45, Keratin complex 2, basic, gene 8; 46, Keratin type II; 47, Krt13 protein; 48, Laminin receptor; 49, Major vault protein (MVP); 50, Microtubule-associated protein, RP/EB family, member 1; 51, Myosin light chain, regulatory B-like; 52, Nucleophosmin 1; 53, p50b; 54, Peroxiredoxin 2; 55, Prolyl 4-hydroxylase, beta polypeptide precursor; 56, Proteasome (prosome, macropain) 28 subunit, alpha; 57, Proteasome alpha 1 subunit; 58, Protein disulfide isomerase associated 6; 59, Protein disulfide-isomerase A3 precursor; 60, Protein synthesis initiation factor 4A; 61, Purine nucleoside phosphorylase; 62, Put. beta-actin (aa 27-375); 63, Rab GDP dissociation inhibitor beta; 64, Rho GDP dissociation inhibitor (GDI) alpha; 65, Rho, GDP dissociation inhibitor (GDI) beta; 66, Serine (or cysteine) proteinase inhibitor, clade B, member 1a; 67, Stathmin; 68, Superoxide dismutase 1, soluble; 69, Superoxide dismutase 1, soluble; 70, Tropomyosin 3, gamma; 71, Tubulin, beta 5; 72, Tyrosine 3/tryptophan 5-monooxygenase activation protein, epsilon polypeptide; 73, Tyrosine 3-monooxygenase/tryptophan 5-monooxygenase activation protein, beta polypeptide; 74, Vacuolar adenosine triphosphatase subunit B; 75, Valosin-containing protein; 76, Vimentin.

**Figure 2:**
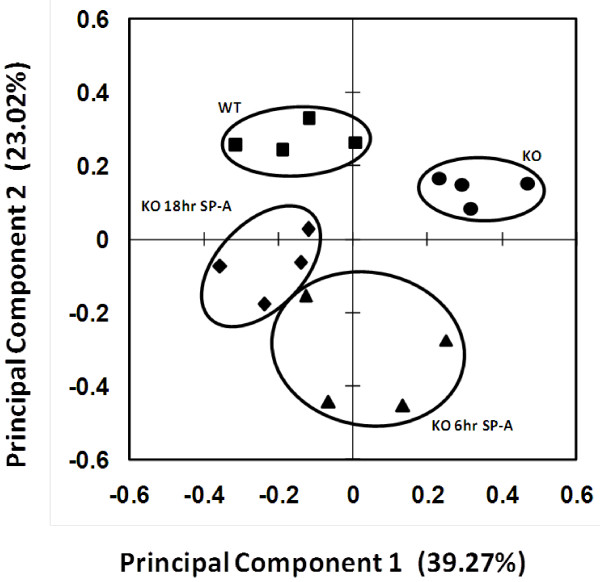
**Principal component analysis**. A plot of the principal component analysis for the 234 significant (ANOVA, p < 0.05) protein spots is shown. The markers represent the weighted average for the first two principal components for the 234 proteins for each individual in each of the groups: SP-A knockout (KO) (●), SP-A knockout treated with SP-A for 6 hr (KO 6 hr SP-A) (▲), SP-A knockout treated with SP-A for 18 hr (KO 18 hr SP-A) (♦), and wild type (WT) (■).

**Figure 3:**
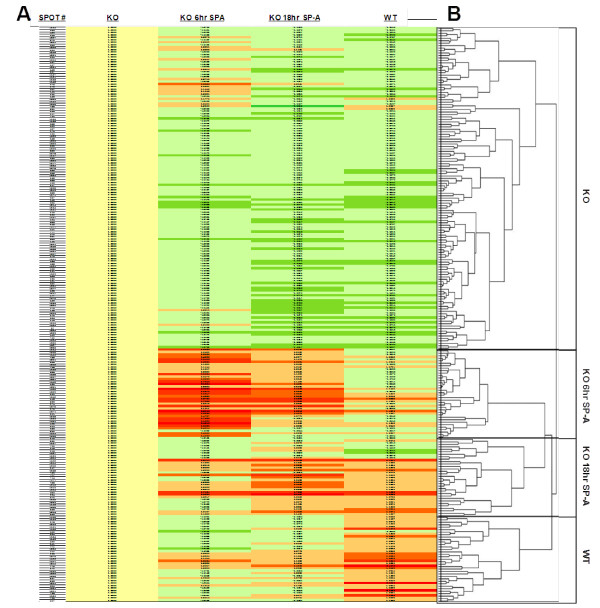
**Heat map and dendrogram of protein spots with significant changes (ANOVA, p < 0.05)**. Panel A. A heat map depicts differences in protein expression from the SP-A knockout (KO). A line representing each numbered protein spot that was found to be significantly different by ANOVA is colored using a red to green scale with dark red indicating the greatest increases and dark green indicating the greatest decreases. Baseline KO is represented by the yellow column on the left. Each of the 234 significant protein spots is indicated by a colored bar. Proteins at levels higher than in the KO mice are in red (dark red > 25% increase; orange < 25% increase). Protein spots with levels lower than in the KO mice are indicated in green (dark green > 25% decrease; light green < 25% decrease). Map columns include: column 1, spot number (#); column 2, SP-A knockout (KO) (columns 3-5 are compared to this value); column 3, differences between KO vs. KO treated with SP-A for 6 hr (KO 6 hr SP-A); column 4, differences between KO vs. SP-A knockout treated with SP-A for 18 hr (KO 18 hr SP-A); and column 5, differences between KO vs. wild type (WT). Panel B. A dendrogram has been generated showing hierarchical clustering of protein spots based on expression profiles derived from the data depicted in the heat map. Each leaf or line on the left of the dendrogram corresponds to the adjacent bar in the heat map. On the dendrogram protein spots with similarities in their expression patterns are grouped together. The labeled groups indicate groups or protein spots that tended to be at their highest level in the indicated group (i.e the group labeled WT protein spots were at higher levels in WT than the other groups), although there were some exceptions (see results). Smaller distances between branches indicate a more similar expression profile. The clustering of the expression profiles of protein spots for this experiment can easily be seen by the division of the significant (ANOVA, p < 0.05) protein spots that are typically at their highest levels in the designated group.

**Figure 4:**
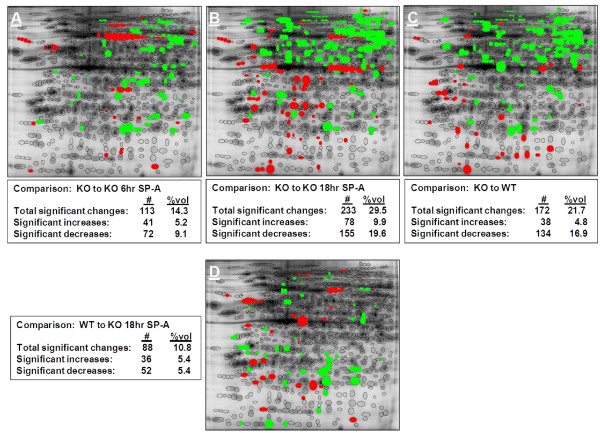
**Gel comparisons**. Locations of spots with significant changes by direct comparisons (t-test, p < 0.05) are mapped to show significant increases in red and significant decreases in green. Comparisons include: **Panel A**, SP-A KO to KO 6 hr SP-A; **Panel B**, KO to KO 18 hr SP-A; **Panel C**, KO to WT; and **Panel D**, WT to KO 18 hr SP-A. The text box with each image lists the total significant changes, significant increases, and significant decreases as both the number of protein spots (#) and percent of total volume (%vol) of resolved protein on the gel.

**Figure 5:**
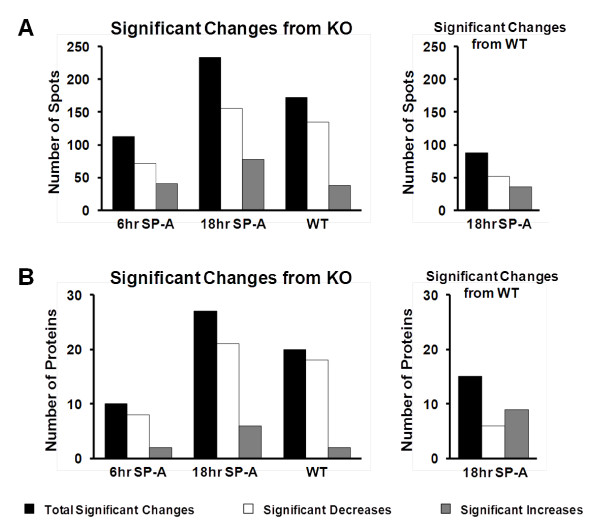
**Summary of significant changes**. In Panel A the graphs depict the number of significant changes in protein spots (from a total of 791) compared to KO (left) or WT (right). The black bar represents the total significant changes, the white bars represent significant decreases, and the gray bars represent significant increases. In Panel B the same changes are shown but in this case the changes evaluated were in the 76 identified proteins.

Following automatic spot detection and manual editing, 791 protein spots were defined and matched across 2D-DIGE gels from all samples and then subjected to further analysis. A reference gel is shown in Figure 1. Details of 2D-DIGE processing and data analysis are provided in Additional Files 1 and 2. When experimental groups were compared, ANOVA (p < 0.05) revealed significant differences in 234 spots, and these were subjected to additional analyses (Figures 2, 3, 4 and 5). The initial analysis was done using the individual spots data. All 791 spots were harvested from a preparative gel and we were able to identify 76 distinct proteins by MALDI-ToF/ToF with confidence intervals > 95% and ProteinPilot scores of > 61. The identified proteins were made up of 191 spots including "nearest neighbor" IDs and accounted for 24.1% of the detectable protein spots and 70.2% of the stained protein on 2D-DIGE gels. On the reference gel in Figure 1 the identified proteins with 191 spots circled are shown. The identified proteins were entered into the Ingenuity Pathways Analysis program to gain further insight into biological processes that might be affected (Table 1). Some of these identified proteins consisted of multiple spots and statistical analysis was done on the combined normalized volume of all of the spots constituting a specific protein in each of our functional groups (Tables 2, 3, 4, 5, and 6). As mentioned above, some of the individual proteins are represented by a single spot and others may be one of multiple spots making up isoforms of a specific protein. Pairwise statistical comparisons between treatment groups were then done for both the individual spots and identified proteins and the results are summarized in Figure 5. Assignment of all identified proteins into subgroups is provided in Additional File 3 and comparisons for all identified proteins are listed in Additional File 4.

**Table 1:** p values for pathways identified by Ingenuity Pathways Analysis.

IPA Top Canonical Pathways	p-value
Regulation of Actin-based Motility by Rho	6.85E-08
RhoA Signaling	3.98E-07
Actin Cytoskeleton Signaling	3.35E-06
Fcγ Receptor-mediated Phagocytosis in Macrophages and Monocytes	3.36E-06
Clathrin-mediated Endocytosis Signaling	6.11E-05
NRF2-mediated Oxidative Stress Response	1.09E-4

**Table 2:** Changes in actin-related/cytoskeletal (ARC) proteins (as compared to KO baseline).

**Gel No**.	Protein Name	KO 6 hr SP-A	KO 18 hr SP-A	WT
1	65-kDa macrophage protein	**↓**	↓*	**↓**
2	Actin related protein 2/3 complex, subunit 5	**↓**	UN	**↑**
3	Actin-related protein 3	↓*	↓*	**↓**
4	Actr2 protein	**↓**	**↓**	**↓**
6	Annexin A2	↓*	↓*	↓*
11	Calpain, small subunit 1	**↓**	↓*	**↓**
12	Capping protein (actin filament) muscle Z-line, alpha 2 (CapZ alpha-2)	**↓**	**↑**	**↑**
13	Capping protein (actin filament) muscle Z-line, beta isoform a (CapZ beta)	↓*	**↓**	**↓**
15	Chaperonin subunit 2 (beta)	**↓**	↓*	↓*
19	Chloride intracellular channel 1	**↓**	↓*	↓*
20	Chloride intracellular channel 4 (mitochondrial)	**↑**	↑*	**↑**
22	Coactosin-like 1	**↑**	↑*	**↑**
24	Eno1 protein (Alpha-enolase)	**↑**	**↓**	**↓**
25	Eukaryotic translation initiation factor 5A	**↑**	**↑**	**↑**
26	Ezrin	**↓**	↓*	↓*
27	F-actin capping protein alpha-1 subunit (CapZ alpha-1)	**↑**	**↑**	**↑**
30	Gamma-actin	**↓**	↓*	↓*
31	Gelsolin precursor	**↑**	**↓**	**↑**
33	Guanine deaminase	**↓**	**↓**	**↓**
34	Heat shock protein 1, beta	**↑**	**↓**	**↑**
39	Hematopoietic cell specific Lyn substrate 1	UN	**↑**	**↑**
45	Keratin complex 2, basic, gene 8	**↓**	**↑**	↑*
46	Keratin type II	**↓**	↓*	**↓**
47	Krt13 protein	**↑**	↑*	**↓**
49	Major vault protein (MVP)	↓*	↓*	↓*
50	Microtubule-associated protein, RP/EB family, member 1	**↓**	↓*	**↓**
51	Myosin light chain, regulatory B-like	**↑**	**↑**	**↑**
53	p50b; Leukocyte-specific protein 1 (LSP1)	**↓**	**↓**	**↓**
62	Put. beta-actin (aa 27-375)	**↑**	↑*	**↑**
63	Rab GDP dissociation inhibitor beta	**↓**	↓*	↓*
64	Rho GDP dissociation inhibitor (GDI) alpha	**↑**	**↓**	**↓**
65	Rho, GDP dissociation inhibitor (GDI) beta	**↑**	**↑**	**↑**
67	Stathmin	**↑**	**↑**	↑*
69	Tropomodulin 3	**↑**	**↑**	**↑**
70	Tropomyosin 3, gamma	**↓**	**↓**	**↑**
71	Tubulin, beta 5	**↑**	**↓**	**↑**
75	Valosin-containing protein	**↓**	↓*	↓*
76	Vimentin	**↑**	**↓**	↓*

	**Total changes**	**21↓, 16↑, 1UN**	**24↓, 13↑, 1UN**	**21↓, 17↑**
	**Total significant changes**	4↓*	13↓*, 4↑*	9↓*, 2↑*

Comparison of mean normalized volumes (see Additional File 4) for proteins from KO mice to KO 6 hr SP-A, KO 18 hr SP-A, and wild-type mice. Increased (**↑**), decreased (**↓**), unchanged (UN), determined to be significant (p < 0.05) by t-test (*).

**Table 3:** Changes in regulation of inflammation (ROI) proteins (as compared to KO baseline).

**Gel No**.	Protein Name	KO 6 hr SP-A	KO 18 hr SP-A	WT
5	Alpha-fetoprotein	↑*	↑*	**↑**
6	Annexin A2	↓*	↓*	↓*
7	Alpha-fetoprotein	**↓**	**↓**	**↓**
16	Chia protein	**↓**	**↑**	**↓**
17	Chitinase 3-like 3 precursor (Ym1)	**↑**	↓*	↓*
18	Chitinase-related protein MCRP	**↑**	**↑**	**↓**
24	Eno1 protein (Alpha-enolase)	**↑**	**↓**	**↓**
25	Eukaryotic translation initiation factor 5A	**↑**	**↑**	**↑**
34	Heat shock protein 1, beta (HSP90AB1)	**↑**	**↓**	**↓**
35	Heat shock protein 5 precursor (GRP78)	**↑**	↓*	**↓**
36	Heat shock protein 65 (HSP60)	**↓**	↓*	↓*
37	Heat shock protein 8 (HSC70, HSC71)	**↑**	**↑**	**↓**
38	Heat shock protein 90, beta (Grp94), member 1	**↓**	↓*	↓*
39	Hematopoietic cell specific Lyn substrate 1	UN	**↑**	**↑**
40	Heme-binding protein	**↓**	**↓**	↓*
42	High mobility group 1 protein	**↑**	**↓**	**↑**
53	p50b; Leukocyte-specific protein 1 (LSP1)	**↓**	**↓**	**↓**
72	Tyrosine 3/tryptophan 5-monooxygenase activation protein, ε	**↑**	**↓**	**↓**
73	Tyrosine 3-monooxygenase/tryptophan 5-monooxygenase activation protein, β	**↑**	**↓**	**↓**
76	Vimentin	**↑**	**↓**	↓*

	**Total changes**	**7↓, 12↑, 1UN**	**14↓, 6↑**	**16↓, 4↑**
	**Total significant changes**	1↓*, 1↑*	5↓*, 1↑*	6↓*

Comparison of mean normalized volumes (see Additional File 4) for proteins from KO mice to KO 6 hr SP-A, KO 18 hr SP-A, and wild-type mice. Increased (**↑**), decreased (**↓**), unchanged (UN), determined to be significant (p < 0.05) by t-test (*).

**Table 4:** Changes in protease balance/chaperone function (PBCF) proteins (as compared to KO baseline).

**Gel No**.	Protein Name	KO 6 hr SP-A	KO 18 hr SP-A	WT
11	Calpain, small subunit 1	**↓**	↓*	**↓**
14	Cathepsin D precursor	**↓**	**↓**	**↓**
15	Chaperonin subunit 2 (beta)	**↓**	↓*	↓*
19	Chloride intracellular channel 1	**↓**	↓*	↓*
21	CNDP dipeptidase 2	↓*	↓*	**↓**
22	Coactosin-like 1	**↑**	↑*	**↑**
24	Eno1 protein (Alpha-enolase)	**↑**	**↓**	**↓**
34	Heat shock protein 1, beta (HSP90AB1)	**↑**	**↓**	**↓**
35	Heat shock protein 5 precursor (GRP78)	**↑**	↓*	**↓**
36	Heat shock protein 65 (HSP60)	**↓**	↓*	↓*
37	Heat shock protein 8 (HSC70, HSC71)	**↑**	**↑**	**↓**
38	Heat shock protein 90, beta (Grp94), member 1	**↓**	↓*	↓*
55	Prolyl 4-hydroxylase, beta polypeptide precursor	↑*	**↑**	↓*
56	Proteasome (prosome, macropain) 28 subunit, alpha	**↓**	**↑**	**↑**
57	Proteasome alpha 1 subunit	**↓**	**↓**	**↓**
58	Protein disulfide isomerase associated 6	**↓**	**↓**	**↓**
59	Protein disulfide-isomerase A3 precursor	**↓**	**↓**	**↓**
66	Serine (or cysteine) proteinase inhibitor, clade B, member 1a	**↓**	**↑**	**↑**
75	Valosin-containing protein	**↓**	↓*	↓*

	**Total changes**	**13↓, 6↑**	**14↓, 5↑**	**16↓, 3↑**
	**Total significant changes**	2↓*	8↓*, 1↑*	6↓*

Comparison of mean normalized volumes (see Additional File 4) for proteins from KO mice to KO 6 hr SP-A, KO 18 hr SP-A, and wild-type mice. Increased (**↑**), decreased (**↓**), unchanged (UN), determined to be significant (p < 0.05) by t-test (*).

**Table 5:** Changes in regulatory/differentiative processes (RDP) proteins (as compared to KO baseline).

**Gel No**.	Protein Name	KO 6 hr SP-A	KO 18 hr SP-A	WT
25	Eukaryotic translation initiation factor 5A	**↑**	**↑**	**↑**
41	Heterogeneous nuclear ribonucleoprotein K	↓*	↓*	↓*
42	High mobility group 1 protein	**↑**	**↓**	**↑**
43	Hnrpf protein	**↓**	**↓**	**↓**
44	Kappa-B motif-binding phosphoprotein	**↓**	**↓**	**↓**
52	Nucleophosmin 1	**↓**	**↓**	**↓**
53	p50b	**↓**	**↓**	**↓**
60	Protein synthesis initiation factor 4A	**↑**	**↓**	↓*

	**Total changes**	**5↓, 3↑**	**7↓, 1↑**	**6↓, 2↑**
	**Total significant changes**	1↓*	1↓*	2↓*

Comparison of mean normalized volumes (see Additional File 4) for proteins from KO mice to KO 6hr SP-A, KO 18hr SP-A, and wild-type mice. Increased (**↑**), decreased (**↓**), unchanged (UN), determined to be significant (p < 0.05) by t-test (*).

**Table 6:** Changes in Nrf2-regulated (NRF) proteins (as compared to KO baseline).

**Gel No**.	Protein Name	KO 6 hr SP-A	KO 18 hr SP-A	WT
1	65-kDa macrophage protein	**↓**	↓*	**↓**
14	Cathepsin D precursor	**↓**	**↓**	**↓**
20	Chloride intracellular channel 4 (mitochondrial)	**↑**	↑*	**↑**
28	Ferritin heavy chain 1	**↓**	↑*	**↓**
29	Ferritin light chain 1	↓*	**↓**	**↓**
30	Gamma-actin	**↓**	↓*	↓*
31	Gelsolin precursor	**↑**	**↓**	**↑**
32	Glucose-6-phosphate dehydrogenase X-linked	**↓**	↓*	↓*
34	Heat shock protein 1, beta (HSP90AB1)	**↑**	**↓**	**↓**
35	Heat shock protein 5 precursor (GRP78)	**↑**	↓*	**↓**
38	Heat shock protein 90, beta (Grp94), member 1	**↓**	↓*	↓*
45	Keratin complex 2, basic, gene 8	**↓**	**↑**	↑*
47	Krt13 protein	**↑**	↑*	**↓**
54	Peroxiredoxin 2	**↓**	UN	**↑**
57	Proteasome alpha 1 subunit	**↓**	**↓**	**↓**
60	Protein synthesis initiation factor 4A	**↑**	**↓**	↓*
62	Put. beta-actin (aa 27-375)	**↑**	↑*	**↑**
68	Superoxide dismutase 1, soluble	**↑**	**↑**	**↓**
71	Tubulin, beta 5	**↑**	**↓**	**↑**
75	Valosin-containing protein	**↓**	↓*	↓*
76	Vimentin	**↑**	**↓**	↓*

	**Total changes**	**11↓, 10↑**	**14↓, 6↑, 1UN**	**15↓, 6↑**
	**Total significant changes**	1↓*	5↓*, 4↑*	6↓*, 1↑*

Comparison of mean normalized volumes (see Additional File 4) for proteins from KO mice to KO 6 hr SP-A, KO 18 hr SP-A, and wild-type mice. Increased (**↑**), decreased (**↓**), unchanged (UN), determined to be significant (p < 0.05) by t-test (*).

#### Principal Component Analysis

When principal component analysis (PCA) was performed on the significantly different spots, the markers for the four gels (each marker represents the gel from one individual animal within an experimental group) were grouped fairly tightly, and each of the experimental groups was well separated from one another (Figure 2). The first principal component accounted for 39.27% of the variance and the second accounted for 23.02%. In each group (KO mice, WT mice, and KO mice treated with SP-A for 18 hr), the four individual points are all rather tightly grouped indicating relatively small variance within each group. The KO mice treated with SP-A for 6 hr are not grouped as tightly indicating greater variance among individual samples as these mice begin responding to SP-A treatment. The relative positions of each group are also informative. A clear separation between WT and KO mice is evident based on principal component 1. However, the KO mice treated with SP-A for 6 hr are quite different from the KO baseline mice and the WT baseline mice. By contrast, at 18 hr after SP-A treatment the mice, based on principal component 1, are in closer proximity to the WT baseline mice than the KO baseline group indicating their overall greater similarity to the WT animals following the SP-A treatment.

#### Heat diagram

A dynamic picture of the changes in expression is afforded by the heat diagram in Figure 3A. The relative expression levels depicted by the heat diagram provide the basis for defining the expression patterns that are summarized by the dendrogram (Figure 3B). In this diagram the KO baseline mice (1st column in yellow) provide the reference values to which the other groups are compared. Each of the 234 significant protein spots is indicated by a colored bar. Proteins at levels higher than in the KO mice are in red (dark red > 25% increase; orange < 25% increase). Protein spots with levels lower than in the KO mice are indicated in green (dark green > 25% decrease; light green < 25% decrease). It is readily apparent that both increases and decreases occur in all of the experimental groups compared to KO mice and, based on the preponderance of the green color in the diagram, most of the proteins are at higher levels in the KO baseline mice. However, a variety of expression patterns can be distinguished. For example, near the top of the diagram there are more than a dozen orange bars in the 6 hr group, indicating an increase over KO baseline levels. In the 18 hr and WT columns most of these have become green, indicating that the increases were transient and that most of the proteins have fallen to levels below those seen in KO baseline mice. In the bottom half of the diagram, there are a number of orange and dark red bars at 6 hr that remain red in the 18 hr SP-A group and in the WT group, indicating a persistent increase in the levels of these proteins after SP-A treatment of KO mice that resembles the WT levels. A number of other patterns are present representing increases and decreases and/or transient and sustained changes. The heat map shows a shift of changes with time after SP-A treatment towards the WT phenotype.

#### Cluster analysis

In Figure 3B the arrangement of the clusters produced by hierarchical clustering of the data depicted in the heat diagram (Figure 3A) is portrayed with a dendrogram in which the 234 significant spots are grouped according to patterns of protein expression as levels of each spot are compared in the various experimental groups. With this arrangement the changes between groups can clearly be seen along the axis of the dendrogram, where protein spots are bracketed together and labeled with the group name where they are typically being expressed at the highest levels. For example, in the WT portion of the dendrogram, each leaf or each line (which represents one protein spot) on the axis is a protein that is typically (but not always) found at higher levels in the WT group compared to any of the other groups. The expression of more than half of the significant spots was highest in the untreated KO mice. The 6 hr SP-A, 18 hr SP-A, and WT groups all have approximately equal numbers of spots reaching their highest levels in each group. There were two major branches in the dendrogram. One represents the untreated KO mice and the other represents the two SP-A-treated KO groups and the WT mice. Within that second branch, the two SP-A-treated KO groups had a common sub-branch and were separated from the WT mice. The overall dendrogram analysis indicates that the KO and WT mice are on different branches and clearly separated from one another, whereas the treated mice share a common branch with the WT and appear to be closer to them than the KO mice. Although, as stated above, spots are typically at their highest abundance in the named category (KO 6 hr SP-A, WT, etc.), there are exceptions. An example of this is seen in the heat map at the top of the KO 6 hr SP-A column where there are a number of spots that are at their most abundant level, as indicated by the orange and red bars. It is also important to note that the KO division of the dendrogram has two major branches. At the top of the dendrogram, the smaller first branch appears to represent many protein spots that are at higher levels in the 6 hr SP-A-treated group, but then apparently (at 18 hr, green bars in heat map) drop to levels below those of the KO mice, suggesting a transient response at 6 hr (as discussed above). These spots could potentially be viewed as more related to the major 6 hr SP-A cluster. The much larger remainder of the KO branch contains multiple subdivisions, but all represent protein spots that are typically at lower levels in the SP-A-treated groups and the WT than in the KO mice.

#### Gel maps of protein changes

Figure 4 shows the reference gel with significant differences in protein spots between groups colored either green for decreases or red for increases. The significant differences are noted as the number of spots (out of a total of 791 spots) that changed significantly, and as the percentage of total volume of protein on the gel that are represented by the significant spots. Total volume is determined by adding the densitometric values of all of the expressed proteins resolved on the gel. Panels A through C of Figure 4 show changes as compared to the KO baseline control animals. In Panel D the protein spots from WT mice are compared to those of the KO mice 18 hr after SP-A treatment.

Comparison of the KO baseline mice to the KO 6 hr animals (Panel A) revealed 113 significant changes (41 increases and 72 decreases) accounting for 14.3% (5.2% increases and 9.1% increases) of the resolved protein. The largest number of significant changes is seen when KO baseline mice are compared to KO 18 hr. There are 233 significant changes with approximately 2/3 being proteins that are at higher levels in the KO mice (Panel B). The 233 protein spots make up 29.5% of the resolved protein on the gel. Of these, 78 are proteins expressed at increased levels and 155 are proteins expressed at decreased levels in the KO 18 hr mice compared to the KO baseline mice. These proteins make up 9.9% and 19.6% of the resolved protein, respectively. When the KO to KO 18 hr gel and the KO to WT gel are compared, the pattern of changes is similar, suggesting that many of the changes in protein expression resulting from the exogenous SP-A treatment are restoring protein levels to those approximating the levels characterizing the WT phenotype. This trend can be further seen when the WT is compared to the KO 18 hr (Panel D). The number of significant changes is markedly reduced, confirming the similarity of these two groups. In this comparison there are only 88 protein spots that differ significantly, making up 10.8% of total resolved protein. Panels A and D each depict a lower number of changes compared to Panels B and C, indicating that the protein expression profiles, in terms of the magnitude and number of changing spots, are closer to KO (after 6 hr of SP-A treatment) and WT (after 18 hr of SP-A treatment) profiles, respectively. This can be better appreciated in Figure 5, Panel A, where the total number of spots changes as well as the numbers of spots increased and decreased in each experimental group compared to KO or WT are graphed. A similar conclusion is reached in Panel B of Figure 5 where the same comparisons are made using the 76 identified proteins rather than the 791 total protein spots.

In Additional File 4 the mean normalized volumes (+/- SD) are given for all 76 identified proteins and significant differences for comparisons for each experimental group between groups that are statistically significant are indicated.

#### Categorization of identified proteins

We used different approaches as done previously [26,42,46] with other types of samples (BAL proteins, plasma proteins) to assess the function of the set of 76 identified proteins and their biological relevance to macrophage function. Valuable insight was provided by analyzing the data with the Ingenuity Pathways Analysis (IPA) program. These analyses identified: 1) regulation of actin-based motility; 2) Rho A signaling; 3) Actin cytoskeletal signaling; 4) Fcγ receptor-mediated phagocytosis; 5) clathrin-mediated endocytosis signaling; and 6) the NRF2-mediated oxidative stress response (Table 1). However, as often occurs with IPA, PANTHER, and similar programs, available information is limited because many published functions/relationships of specific proteins have not yet been included and an organ/tissue-specific context is frequently lacking. Another frequent challenge is that functions are sometimes subdivided into so many subgroups that the overall significance is diminished. For these reasons, we also employed a manual curation approach, emphasizing findings from the literature related to the lung and macrophages, where available. This approach, as did IPA, strongly implicated motility, phagocytosis, actin signaling, and endocytosis (which we retained as a single functional group of "actin-related/cytoskeletal proteins" rather than dividing it into 4 subgroups) as the most involved cellular processes and included 37 of the 76 proteins identified in our study (Table 2). Given the role of the alveolar macrophage as a mobile phagocyte, this was anticipated and the identification of a substantial subset of identified proteins involved in these processes indicates that SP-A plays a pivotal role in these macrophage functions.

Other major processes implicated by our protein list included regulation of inflammation (19 proteins) (Table 3), protease balance/chaperone function (18 proteins) (Table 4), regulatory/differentiative processes (8 proteins) (Table 5), and Nrf2-regulated proteins (21 proteins) (Table 6). All proteins and the functional groups they were assigned to are listed in Additional File 3[47-115]. These functional groups also represent important facets of alveolar macrophage biology and thereby constitute a valuable tool in assessing macrophage function in the presence or absence of SP-A. Protease/chaperone function may be very important in the repair of damage to lung tissue and proteins potentially resulting from exposure to noxious material or pathogens and other danger signals. Similarly, the regulation of inflammatory processes is extremely important for innate immune processes, with dysregulation of inflammation playing a central role in many pulmonary disease processes. Finally, the profound differences between circulating blood monocytes and the alveolar macrophage [43,116,117] and between macrophages that have undergone different modes of activation [116,118] indicate the presence of an active regulatory mechanism directing the differentiation of macrophages from monocytes and their activation in various directions.

In addition to the functional categories described above, 21 of the identified proteins (~15-20) have been reported to be regulated by Nrf2. All of these have been reported to either contain antioxidant response elements (ARE) in their genes, or have been demonstrated to be Nrf2-regulated. We did not include proteins that may interact with Nrf2, but are not regulated by it. Nrf2, and its inhibitor, Keap1, are instrumental in regulating proteins that contain an ARE in their promoter regions and have been shown to regulate many antioxidant proteins, a number of cytoprotective enzymes (including Phase 2 detoxifying enzymes), as well as a number of heat shock proteins/chaperones. These proteins help maintain redox balance in the lung by limiting the effects of exogenous and endogenous reactive oxidant species. A number of the identified proteins fall into these categories. In some cases (ferritin light and heavy chain, glucose-6-phosphate dehydrogenase, peroxiredoxin, superoxide dismutase, valosin-containing protein, *et al*.) the presence of an ARE in their genes has been documented, confirming this potential regulatory mechanism. However, many other proteins that we identified have functions such as those listed above that are typically viewed as Nrf2-regulated. Of significant relevance, Nrf2/Keap1 activity can be regulated by interaction with actin filaments [119], adding another dimension to the biological relevance of the changes occurring in the many actin-related/cytoskeletal proteins we identified in the present study.

### Cell area and F-actin measurements after SP-A treatment

**Figure 6:**
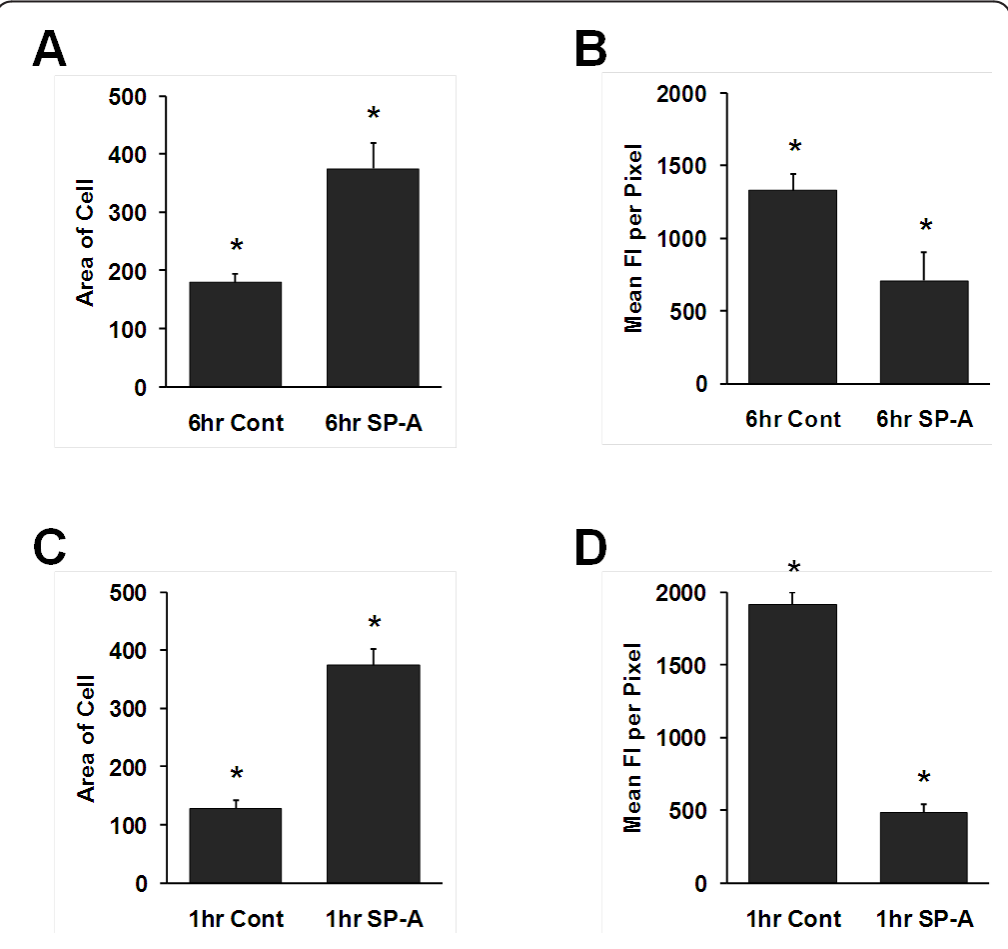
**Determinations of macrophage cell area and F-actin measurements after SP-A treatment**. SP-A KO alveolar macrophages were isolated, cultured overnight, and treated with SP-A (or its control). The cells were then fixed, permeabilized, stained with Alexa Fluor 488 phalloidin, and analyzed as described. A) Cell area is indicated for control and SP-A-treated macrophages after 6 hr of SP-A treatment. The data shown represent measurements of cell area made on cells (n = 115 cells for control; n = 102 cells for SP-A-treated) from 5 mice. T-test indicates a significant difference (*) between groups (p = 0.0016). B) F-actin levels are represented by mean fluorescence intensity/pixel. The data were obtained from analysis of the same number of mice and cells indicated in Panel A. The two groups were significantly different (*; p = 0.016). C) Cell area was determined from macrophages after one hour of SP-A treatment. Control values (n = 60 cells) and SP-A treated values (n = 62 cells) were significantly different (*; p < 0.0001). D) F-actin levels in the samples described in Panel C, as represented by mean fluorescence intensity/pixel, were significantly different (*; p < 0.0001).

Given the relatively large number of actin-related/cytoskeleton proteins that changed in response to SP-A, as "proof of principle" we measured cell size and levels of F-actin. In macrophages that were treated with SP-A for 6 hr and stained with Alexa Fluor 488 phalloidon we examined cell area (Figure 6A) and the mean fluorescence intensity/pixel (Figure 6B) as a measure of the amount of F-actin/cell. Data were acquired from > 100 cells obtained from 5 mice. Analysis of cell size revealed that control macrophages from SP-A KO mice were significantly (p = 0.0016) smaller than macrophages from the same mice that were treated *in vitro *with SP-A for 6 hr. The mean fluorescence intensity/pixel in cells after SP-A treatment for 6 hr was significantly (p = 0.016) reduced to approximately 1/2 of that seen in the control cells.

Macrophages treated with SP-A for 1 hr and their controls (Figure 6C, D), the cell size and the mean fluorescence intensity/pixel exhibited similar changes to those seen at 6 hr. In fact, at the 1 hr time point (a time point used in several studies in which we studied phagocytosis as a biological readout [27,29]) cell size increased three-fold after SP-A treatment and the mean fluorescence intensity/pixel decreased almost four-fold. Both differences were highly significant (p < 0.0001; p < 0.0001).

## Discussion

The absence of SP-A in KO mice has been shown to increase susceptibility to infection, compromise bacterial clearance and survival from pneumonia, and adversely affect the response to various other types of lung injury [26,28,33,36-42,120]. However, virtually nothing is known about the impact of SP-A on the alveolar macrophage proteome under basal or unstimulated conditions. We employed a discovery proteomics approach to investigate, *in vivo*, the impact of SP-A on global protein expression by the alveolar macrophage as a way of gaining insight into molecules and mechanisms affected by SP-A under basal conditions. We thus studied and compared the intracellular proteome of the alveolar macrophage from WT mice and SP-A KO mice, and SP-A KO mice "rescued" with exogenous SP-A and sacrificed at 6 hr, a time point we have previously shown to be sufficient for the same dose of exogenous SP-A to restore tubular myelin formation in KO mice [121], and 18 hr after treatment. In the present study under basal conditions we observed: 1) differences in individual protein content between macrophages from KO and WT mice; 2) that a single *in vivo *treatment of KO mice with SP-A exerted a significant impact on the expression of proteins by alveolar macrophages compared to untreated KO mice; 3) as the time after SP-A treatment of KO mice increased, the proteomic profile more closely approximated that of WT mice; 4) a diverse set of SP-A-dependent functions in the alveolar macrophage, some of which have not been identified *in vivo *previously; including proteins involved in the regulation of the cytoskeleton; and 5) changes in F-actin and cell size of the alveolar macrophage in response to SP-A.

In previous studies of BAL proteome analysis, SP-A was found, under both resting conditions and after various perturbations (infection, ozone exposure), to have a major impact on BAL protein expression, particularly with respect to proteins involved in host defense or the regulation of redox balance [26,42]. Proteins secreted by the alveolar macrophage, and by other lung cells (particularly epithelial cells), together with proteins from plasma, are the principal sources of BAL protein. The observations made here indicate that the alveolar macrophage may also be a significant contributor to the observed BAL changes. Furthermore, the observed alveolar macrophage proteome changes in response to SP-A may contribute to the SP-A-dependent differences in the phagocytic function of these cells, which may in turn contribute to differences in disease susceptibility. In fact, it has been shown that both the phagocytic activity of the alveolar macrophages from SP-A KO mice [28], and the survival of SP-A KO mice were compromised compared to WT mice following infection with *K. pneumoniae *in the presence or absence of ozone-induced oxidative stress [27,28]. The present findings, as assessed by the multitude of molecules and pathways affected by SP-A, support the notion that the alveolar macrophage from the KO mice is not biologically "ready" to successfully combat injurious agents, and hence the poorer clinical outcome shown in studies of the SP-A KO mice.

The SP-A dose and time points, as noted above, were based on a study where using the same conditions we were able to restore tubular myelin to the lungs of KO mice [121]. Moreover, based on measurements of SP-A levels in the BAL of lungs of C57BL/6 mice, the dose used was within the physiological range. A similar exogenous SP-A treatment has been reported [3,122], although a number of other studies have used much higher doses [20,123-125] that are clearly supraphysiological.

The "restoration" of the global pattern of protein synthesis in KO macrophages to that resembling WT macrophages, when exogenous SP-A was given, was remarkable. In 59 out of the 76 (78%) identified proteins the change seen, compared to KO at 18 hr after exogenous SP-A, was consistent with the differences between KO and WT. In other words, when WT levels were lower than KO, the 18 hr SP-A value was lower than KO and the opposite trend was apparent when WT levels were greater than KO. However, this transition from KO towards WT in response to SP-A treatment was complex, because levels of some proteins (~1/3) increased and others (~2/3) decreased in WT macrophages. This raised the possibility that with different proteins SP-A may influence protein synthesis, metabolism, and/or secretion by macrophages to achieve the intracellular levels appropriate for baseline levels in the macrophage.

Changes in the proteome of the treated versus untreated mice could be the result of several different regulatory mechanisms. SP-A may act directly on the alveolar macrophage, through different receptors to which it is known to bind [13,126-128], to directly influence the levels of expression of specific proteins. A second potential mechanism would involve indirect effects of SP-A on macrophage protein expression that result from the direct influence of SP-A on other cells (such as the type II cell), causing the synthesis and release of mediators that, in turn, regulate the production of specific proteins by the alveolar macrophages in a paracrine fashion. A third possibility is that SP-A exerts its influence on the alveolar macrophage proteome by regulating the release of regulatory molecules from one set of alveolar macrophages that act on themselves or another set of alveolar macrophages via either autocrine or paracrine mechanisms, respectively. Regardless of whether one or more of these possibilities contribute to the proteome changes observed after SP-A treatment, what is clear is that SP-A, either via its direct or indirect effects, has a major impact on the protein expression pattern of the alveolar macrophage. This impact includes significant changes in more than half of the identified proteins.

An important indirect factor in terms of SP-A regulation of gene expression becomes apparent when we consider one of our previous studies of the BAL proteome of KO mice in which we postulated increased oxidative stress in the absence of SP-A [26]. In the current study, this would likely be manifested by changes in the expression of Nrf2-regulated proteins. Indeed, the Nrf2-mediated oxidative stress response pathway was identified as a significant pathway in the present study. Consistent with this notion are the observations made here where nearly 1/3 of the identified proteins are Nrf2-regulated, and are expressed at higher levels in KO than in WT mice. In the WT and with SP-A treatment, levels of expression of many of these proteins are significantly reduced, suggesting a lessening of oxidative stress. Glucose-6-phosphate dehydrogenase, a regulator of oxidative stress [129] stands out as an example of this. This enzyme is expressed at significantly higher levels in KO than in WT mice and the 18 hr SP-A treatment reduces it to levels significantly lower than KO baseline.

The most common phenotypic trait that has been examined with the SP-A KO mice is increased susceptibility to pneumonia. The simplest explanation for this phenotype is that host defense against specific pathogens is compromised in the absence of SP-A and its opsonic function. Indeed, replacement of SP-A in these models has afforded some restoration of normal host defense function by apparently increasing pathogen clearance [39,130]. Additional studies in which exogenous SP-A has been administered to SP-A KO mice have shown a diversity of functions or regulatory mechanisms being restored. These include SP-A effects on the regulation of allergic or inflammatory responses [3,120,122,131,132] and other host defense molecules [20,125], as well as the ability of exogenous SP-A to restore tubular myelin, an extracellular form of surfactant [121]. This diversity of effects, along with our BAL proteomic studies [26,42], indicate that SP-A exerts a pleiotropic effect in lung biology and/or health. The observations from the present study not only provide support for an SP-A pleiotropic effect on lung biology, but identify many protein molecules and pathways affected by the interaction of SP-A with the alveolar macrophage. Together these put forward the notion that the interaction of SP-A with the alveolar macrophage under basal conditions is critical, in terms of preparing and maintaining the alveolar macrophage in a state of "readiness" so that it can potentially successfully combat injurious agents. Furthermore, based on the findings of the present study where SP-A exhibits a broad and varied impact on the alveolar macrophage proteome and previous studies of the BAL proteome where the baseline expression profile of SP-A KO and WT mice differed significantly [26,42], we postulate that these differences are key to underlying mechanisms that are responsible for the less favorable clinical outcome of the SP-A KO mice with pneumonia [28,39,40,131] or bleomycin-induced lung injury [120] where SP-A may be dysfunctional due to its being oxidized. Thus, the collective evidence of the role of SP-A in the first line defense of the alveolar surface depicts a diverse array of SP-A-mediated functions that help maintain lung health, or attenuate injury when the lung is challenged with various injurious agents, irritants, pathogens, and toxins present in the external environment. Given the constant exposure of the distal lung surface to all these external harmful agents, it is important that mechanisms be in place to remove these potentially damaging agents and maintain normal lung structure. The alveolar macrophage and SP-A, both important components of innate immunity, appear to play key roles in insuring that this occurs.

SP-A has previously been shown to exert regulatory effects on the production of several macrophage proteins [7,14,16-21,125]. The present study shows for the first time that an acute *in vivo *treatment with SP-A under unstimulated, baseline conditions affected the levels of significantly more and diverse macrophage proteins than previously known. These include proteins involved in macrophage motility, phagocytosis, neutralization of reactive oxidant species and other toxins, removal of damaged tissue/proteins from the alveolus, and other functions. These effects begin to be evident by as early as 6 hr after exogenous SP-A treatment. By 18 hr after intratracheal SP-A treatment, the alveolar macrophage proteome of the KO mouse becomes strikingly similar to that of WT animals that have been continually and naturally exposed to SP-A. The diversity of changed proteins broadly indicates that SP-A, under basal conditions, plays a major role in alveolar macrophage "health" in terms of its potential ability to move about and its "readiness" for pathogen elimination and tissue repair.

The responses identified exhibited several consistent patterns of protein expression. In one pattern the KO mice and WT mice differed significantly from one another (both increases and decreases) and intermediate responses proceeding in the same direction were observed at the two SP-A replacement time points. This pattern was observed in 17 of the 20 proteins in which KO and WT differed significantly (see Additional File 4). The obvious conclusion from this subset of responses is that lack of continually available SP-A in the KO mice alters protein expression and that acute administration of exogenous SP-A leads toward a restoration of the WT phenotype. Of the total of 76 proteins identified in the present study, 37 were proteins with functions broadly related to actin and the cytoskeleton, and of these 37 proteins, 11 differed significantly between KO and WT and followed the pattern described above where SP-A treatment changes expression levels toward those seen in WT. Many other proteins in the actin-related/cytoskeletal group (n = 21 of 37) exhibited the same trend without achieving statistical significance. In most of the 32 responses made up by these two groups (significant, n = 11 + not significant but with a similar trend, n = 21), the time points after SP-A replacement showed a logical progression from KO to WT, indicating a trend toward restoration of the WT phenotype by exogenous SP-A treatment. The inclusion of so many of the significantly changing proteins (n = 11) in the actin/cytoskeleton-related protein group out of 20 significant changes between WT and KO indicates that SP-A has a profound influence on actin-related/cytoskeletal processes such as macrophage motility and phagocytosis, potentially explaining the susceptibility of the KO mice to injury or infection. *In vitro *studies have shown that an intact actin cytoskeleton, as assessed by the use of cytochalasin D, an actin depolymerizing agent, is required for certain SP-A-mediated processes [44] and in extrapulmonary tissues SP-A regulates F-actin filament organization [45]. Although given the importance of actin in cell motility, phagocytosis, and endocytosis, it is not surprising that changes in these proteins do occur, but the extent to which SP-A affects the content of so many actin/cytoskeletal-related proteins *in vivo *is remarkable. Significant changes in protein content with a similar pattern were also seen in several proteins involved in the inflammatory regulation (n = 4) and protease/chaperone (n = 5) functional groups.

The SP-A-dependent changes in actin-related/cytoskeletal proteins may also be the nexus for the differences in the other functional groups. As mentioned earlier, many of the changing proteins are either known to be regulated by Nrf2 or belong to functional classes known to be Nrf2-regulated [133]. Keap 1, the repressor of Nrf2, has been shown to regulate Nrf2 activity through its binding to the actin cytoskeleton [119]. Similarly, a role for the actin cytoskeleton has been described for NF-κB [134], a mechanism via which SP-A exerts some of its functions [44,135,136]. These two transcriptional regulators in addition to each interacting with the actin cytoskeleton, respond in concert to some stimuli in various cells or tissues and a number of genes regulated by both of them [137]. However, the interaction between NF-κB and Nrf2 is complex and may vary in different systems or under different circumstances [138].

In addition, a number of proteins (n = 13) differed significantly between KO mice and mice that had received SP-A 18 hr earlier, even though no significant differences were observed between KO and WT mice. For those proteins however, in most cases the trend in the WT usually resembled that of the effect seen 18 hr after SP-A. This subset of responses supports a scenario where a robust acute response to SP-A is observed, with some of the proteins exhibiting an overcompensation of expression. For example, a number of protein spots (Figure 5) are increased at 6 hr but decreased at 18 hr after treatment, as in the WT, although they may not have not reached significance yet, indicating that some protein changes need a longer exposure to SP-A and/or multiple doses of SP-A to reach the WT phenotype. These responses included 6 proteins from the actin group and 3 from the protease/chaperone group, indicating that the acute SP-A treatment (like the continuous presence of SP-A in WT) has a significant potential to contribute to alterations in macrophage function.

Although statistical techniques such as gene set enrichment analysis or hypergeometric distribution calculations have been used with microarray data to determine whether changes in subsets of genes differ significantly from a reference group or the overall population in a specific group, these cannot be used here. This is because of the relatively small data set examined by gel-based proteomics and the comparatively large numbers of proteins in our subsets that make up a substantial percentage of our overall population or reference group. For example 50% of the identified proteins in our reference group were in our actin-related group and 28% were included in the Nrf2-related proteins, thereby violating the assumption that the analyzed groups be independent of one another and precluding the use of such methods in this study [139,140].

The large number of actin-related proteins with altered levels of expression after SP-A treatment led us to speculate that we would be able to validate the proteomic findings with a preliminary examination of parameters related to actin cytoskeletal dynamics. In fact, macrophages from SP-A KO mice treated with SP-A *in vitro *were significantly larger than comparable control cells and had lower amounts of F-actin, both at the 6 hr time point used for this proteomic study (although total gamma-actin levels were unchanged at this time point) and at 1 hr, a time point where we have previously demonstrated an SP-A-dependent enhancement of phagocytosis by alveolar macrophages [29]. As SP-A stimulation increased cell size, redistribution of the cortical actin over a larger area likely contributes to the decrease in the actin intensity per pixel. We speculate that decreases in F-actin reflect cells that are more actively remodeling their cytoskeletons, resulting in cells of increased size. The larger size may indicate the availability of adequate amounts of excess membrane for phagocytic or endocytic events to occur and the changes in F-actin distribution likely reflect more dynamic actin filaments with altered rates of actin polymerization/depolymerization and branching. Thus, in the absence of SP-A, macrophages appear to be small and "stiff", limiting their motility and phagocytic ability. However, when SP-A is made available, in as little as one hour a more dynamic actin cytoskeleton allows for enhanced motility and ability to successfully locate and clear particles and pathogens. Our previously published observations on *in vivo *comparisons of SP-A KO and WT mice in a model of pneumonia induced by *Klebsiella pneumoniae *[28] and on phagocytosis by alveolar macrophages *in vitro *support this scenario [29].

This study was designed to allow us to study restoration of the WT phenotype from KO alveolar macrophages after giving a single dose of SP-A and studying its effect 6 hr and 18 hr later. Remarkably, the dose and sampling times chosen restored levels of many of the proteins we studied. It is likely that additional doses and time points will further restore the phenotype by regulating the expression of additional proteins with different kinetics of translation and secretion. It is also highly likely that SP-A regulation by continually present SP-A (as in the WT state) or by multiple doses of SP-A will differ somewhat from the pattern resulting from a single acute dose, because in those cases a steady-state equilibrium of the SP-A effects on the alveolar macrophage proteome may be attained. Moreover, we had previously proposed that SP-A "primes" the alveolar macrophage [141] to prepare it to effectively respond to infectious or toxic agents. The proteomic data presented here support a role for SP-A under baseline conditions to drive an ill-prepared "dysfunctional" alveolar macrophage from an SP-A KO mouse towards readiness and effective host defense as afforded by WT alveolar macrophages. Moreover, these data demonstrate the potential for the therapeutic use of a single dose of exogenous SP-A to counter challenges due to injury or infection.

## Conclusions

The observations in the present study show for the first time that an acute *in vivo *treatment with SP-A affects, under basal or unstimulated conditions, the expression of diverse groups of proteins in the alveolar macrophage. The group of actin-related/cytoskeletal proteins appeared to be a prominent one, indicating a significant *in vivo *role of SP-A in alveolar macrophage motility and related functions. This was further substantiated by experiments showing significant changes in cell size and cortical F-actin distribution 1 and 6 hr after SP-A treatment. Furthermore, a significant number of the changed proteins are likely to be Nrf2-regulated indicating a role of SP-A in oxidative stress. We postulate that the SP-A-mediated expression profile of the alveolar macrophage places it in a state of "readiness" to successfully combat various injurious insults. Published comparison studies of SP-A KO and WT mice that showed a poorer outcome in KO in response to various injurious agents support the concept of "readiness." Therefore, SP-A appears to be a critical factor for the "priming" and/or "readiness" of the alveolar macrophage to carry out its innate immune functions effectively and ensure lung health.

## Materials and methods

### Animals

This study was conducted using pathogen-free male WT and SP-A KO male mice on the C57BL/6 genetic background. WT mice were obtained from Jackson Laboratories (Bar Harbor, ME). Breeder pairs of SP-A KO mice had been obtained from Dr. Samuel Hawgood at the University of California, San Francisco and were propagated and raised under specific pathogen-free conditions in a barrier facility at the Penn State College of Medicine [142]. The SP-A KO mice and sentinel mice housed in the same room showed no evidence of respiratory pathogens. The Institutional Animal Care and Use Committee at the Penn State College of Medicine approved this study.

A total of 16, 25-34 g C57BL/6 WT and SP-A KO mice were used to complete the proteomics study. These were divided into four groups with 4 animals per group: 1) SP-A KO control (baseline) mice that did not receive any treatment; 2) SP-A KO mice that were treated with SP-A and sacrificed 6 hr after treatment; 3) SP-A KO mice that were treated with SP-A and sacrificed 18 hr after SP-A treatment; and 4) WT control (baseline) mice that did not receive any treatment.

### SP-A preparation

SP-A was purified from the BAL fluid from normal human lungs obtained from organ donors. The protocol was approved by the Penn State College of Medicine Institutional Review Board. Donor lungs were lavaged with 0.9% saline and the lavage fluid collected and centrifuged at 150 × g for 10 min at 4°C to obtain cell-free BAL. The SP-A was then purified by repeated precipitation with 5 mM calcium chloride. The purity of the preparation was checked by 1D-PAGE with silver stain and by Western blot and determined to be greater than 99 percent pure. LPS content of the SP-A preparation was measured with the QCL-1000 Limulus Amebocyte Lysate assay (Lonza, Walkersville, MD). The LPS content of a 1 μg sample of SP-A was below the detectable limit of the assay (0.1 EU/ml) or < 500 fg/μg of SP-A.

### Treatment of mice with SP-A

Animals were anesthetized with an intramuscular injection of a mixture of Ketamine HCl (Ketaject, Phoenix Pharmaceuticals Inc., St. Joseph, MO) and Xylazine (XYLA-JECT, Phoenix Pharmaceuticals Inc., St. Joseph, MO). Mice were suspended from their incisors and intrapharyngeally instilled with 5 μg of normal human SP-A in 50 μL of 0.9% sodium chloride containing 2 mM calcium chloride. Mice were closely watched to ensure that the entire dose was aspirated. We selected a dose of 5 μg of SP-A/mouse based on our SP-A determinations of total BAL SP-A from C57BL/6 mice (mean 3.2 μg; n = 9). We have previously demonstrated that using this dose of SP-A and a 6 hr time point, we were able to detect tubular myelin in the BAL of SP-A KO mice [121].

### Collection of alveolar macrophages

Mice were anesthetized and subjected to BAL at intervals of 6 hr and 18 hr following treatment with SP-A. We chose 6 hr as our initial time point because we used it successfully in a previous study examining tubular myelin formation [121]. In addition, we postulated that this time interval would be sufficient for new protein synthesis to occur in response to SP-A treatment. The 18 hr time point was chosen to determine the longer term effects of a single dose of SP-A, that could potentially include the consequences of the indirect effects of SP-A. BAL fluid was obtained by instilling PBS, 1 mM EDTA into the lungs through a tracheal cannula using a volume equal to 80% of lung vital capacity (5 × with 0.5 ml of PBS, 1 mM EDTA) for a total of 2.5 ml. For each of the five washes, the fluid was instilled and withdrawn 3 times with chest massage during withdraw. The BAL fluid was then centrifuged at 150 × g for 5 min at 4°C and the cell pellet washed with 1 mL of PBS, 1 mM EDTA. Total cell counts were performed using a hemocytometer and cytocentrifuge preparations done to obtain differential cell counts. The washed cells were then centrifuged again and the pellets frozen at -80°C for subsequent proteomic studies.

### Preparation of samples for 2D-DIGE

Frozen macrophage pellets were lyophilized until complete dryness and resuspended in 25 μL of standard cell lysis buffer (30 mM TrisHCl, 2 M thiourea, 7 M urea, 4% CHAPS, pH 8.5). Protein determinations were done using the Bio-Rad Protein Assay (Bio-Rad, Hercules, CA) and the concentration of protein was adjusted to 1 mg/ml for CyDye labeling.

### 2D-DIGE labeling (minimal labeling) and electrophoresis for 2D-DIGE

We have used this basic technique in previous studies for other types of protein samples [26,46,143] but a detailed account, including a number of modifications and refinements appears below. Information about the 2D-DIGE study is provided in a form that complies with the most recent version of Minimum Information About a Proteomics Experiment-Gel Electrophoresis (MIAPE-GE) standards currently under development (<http://www.psidev.info/miape/MIAPE_GE_1_4.pdf >) by the Human Proteome Organization Proteomics Standards Initiative (see Additional File 1). Two sets of samples from each group were prepared for minimal labeling with CyDyes. Samples from each group were randomly assigned to Cy3 or Cy5 to ensure no dye-based artifacts in quantitation. A 20 μg aliquot of macrophage proteins from each sample was labeled with either Cy3 or Cy5 (200 picomoles). A normalization pool was created by combining equal amounts of protein from every sample (16 samples) and an aliquot of the pool was labeled with Cy2 (200 picomoles/20 μg). Equal amounts (20 μg) of Cy3-labeled sample, Cy5-labeled sample, and Cy2-labeled pool samples were mixed and applied to each gel. An equal volume of 2X sample buffer (2 M thiourea, 7 M urea, 2% IPG buffer (pH 4-7) and 1.2% DeStreak reagent) was added to all samples to give a final volume of 140 μl. Twenty-four cm, pH 4-7 gradient Immobiline DryStrips (GE Healthcare) were rehydrated for 18 hr with 450 μl of rehydration buffer (DeStreak™ Rehydration Solution containing 0.5% IPG buffer (pH 4-7). Proteins were subjected to isoelectric focusing on rehydrated strips using an Ettan IPGphor 3 cup loading manifold (GE Healthcare) following the manufacturer's instruction at 20°C and under mineral oil to prevent evaporation. Proteins were focused using the following voltages and times: 3 hr at 300 V (step and hold); 7 hr at 1000 V (gradient); 4 hr at 8000 V (gradient); 4 hr at 8000 V (step and hold). After isoelectric focusing the IEF strips were equilibrated in equilibration solution-1 (50 mM TrisHCl, 6 M urea, 30% glycerol, 2% sodium dodecyl sulphate (SDS), 0.5% dithiothreitol) and equilibration solution-2 (50 mM TrisHCl, 6 M urea, 30% glycerol, 2% SDS, 4.5% iodoacetamide) for 15 min, respectively, and then applied to 10% polyacrylamide gels (26 cm-w × 20 cm-h × 1 mm thick), sealed with 0.5% low melting point agarose containing bromophenol blue in a buffer of 1X Tris/glycine/SDS buffer (25 mM Tris, 192 mM glycine, 0.1% (W/V) SDS, pH 8.3). Proteins were then separated on the basis of molecular weight using the Ettan DALTtwelve system (GE Healthcare) at 5 W/gel for 30 min and then at 15 W/gel at 20°C until the bromophenol blue dye front was 0.5 cm from the bottom of the gel (~4 hr).

For the preparative (picking) gel an aliquot of 450 μg of sample was diluted with an equal volume of 2X sample buffer (2 M thiourea, 7 M urea, 2% IPG buffer (pH 4-7) and 1.2% DeStreak reagent) and then brought up to a volume of 450 μl with rehydration buffer (DeStreak™ Rehydration Solution and 0.5% IPG buffer (pH 4-7)). Proteins were focused using the following voltages and times: 14 hr at 0 V (passive rehydration); 6 hr at 30 V (active rehydration); 3 hr at 300 V (step and hold); 3 hr at 600 V (gradient); 3 hr at 1000 V (gradient); 3 hr at 8000 V (gradient); 4 hr at 8000 V (step and hold). The strip was then equilibrated as described above and applied to a 10% polyacrylamide gel (26 cm-w × 20 cm-h × 1 mm thick). For the preparative picking gel a single plate for the gel plate sandwich was treated with Bind-Silane solution (80% ethanol, 0.02% glacial acetic acid, 0.001% Bind-Silane) and had reference marker stickers placed on it. After the completion of electrophoresis, the plate that had not been silane-treated was removed from the sandwich and the gel fixed overnight with 30% ethanol, 7.5% glacial acetic acid. The preparative picking gel was then stained with Deep Purple Total Protein Stain (GE, Healthcare) for 2 hr.

### Gel imaging, image analysis, and statistics

Information about the acquisition and processing of data from the 2D-DIGE studies are provided in the form that complies with the most recent version of the guidelines established for Minimum Information about a Proteomics Experiment-Gel Informatics (MIAPE-GI) currently under development by the Human Proteome Organization Proteomics Standards Initiative http://www.psidev.info/files/miape-gi-v1.pdf (see Additional File 2). All two-dimensional gels were imaged on a Typhoon 9410 fluorescent imager (GE Healthcare) at a resolution of 100 μm. Photomultiplier tube voltages were individually set for each of the three colored lasers to ensure maximum, linear signals. The same voltages were used for all the gels. The DIGE gels were imaged at three different wavelengths (Cy2: 520 nm; Cy3: 580 nm; Cy5: 670 nm) and the Deep Purple Total Protein Stain-stained gels were imaged at 100 μm with a separate filter (610 nm).

Gel images were imported into the Progenesis SameSpots v4.0 program (Nonlinear Dynamics) for analysis and their quality assessed by the program. Image quality control with Progenesis SameSpots v4.0 involves checking images for bit depth, color, manipulation prior to analysis, proper file type, saturation, low dynamic range, and stretched contrast. A reference gel with minimum distortion and streaks was selected from the Cy2 gels. Gel alignment was conducted automatically and then checked manually to ensure correct alignment. Spot detection and spot matching across all the gels were conducted automatically, and then spot matching was checked and manually edited to ensure correct matching. Data from all the spots included in analysis were transported to Progenesis PG240 module of the Progenesis SameSpots v4.0 software for further analysis. Statistical analysis was performed by ANOVA and by t-test to confirm the level of significance among various groups. Differences were considered statistically significant with p < 0.05. Principal components analysis (PCA) was performed and dendrograms plotted in the Statistics module of Progenesis SameSpots v4.0 for the further analysis of the patterns of protein expression and protein clustering among groups.

For identified proteins having multiple isoforms, the normalized volumes of all isoforms of a given protein were added together and statistical analysis was performed on the totals using Microsoft Excel.

### Protein identification by mass spectrometry

We have used this procedure in previous studies for other types of protein samples [26,46,143] but a detailed account, including many modifications and refinements appears below. For identification of spots, all 791 protein spots from the experiment were picked from the picking gel using a robot-directed spot picker (Ettan Spot Picker, GE Healthcare). The picker head was calibrated using the reference stickers placed on the preparative picking gel and the gel was picked and gel plugs placed in bar-coded 96 well plates. All gel plugs were washed twice with 200 μl of 200 mM ammonium bicarbonate, 40% acetonitrile for 30 min at 37°C, lyophilized to complete dryness and stored desiccated at 4°C until processing. Plugs selected for processing were rehydrated with 20 μl of 0.02 μg/μl trypsin (Trypsin, proteomics grade, Sigma, St. Louis, MO) for one hour at room temperature. The trypsin solution was then removed and replaced with 50 μl of 40 mM ammonium bicarbonate, 9% acetonitrile and incubated overnight at 48°C. The overnight incubation solution was transferred to new 96-well extraction plates and the plugs extracted with 50 μl of 0.1% trifluoroacetic acid (TFA) for 30 min at 37°C, followed by a second extraction with 50 μl of dH_2_O for 30 min at 37°C. All of the extracts for each plug were pooled and then dried by lyophilization. Dried extracts were then brought up in 200 μl of dH_2_O and dried by lyophilization for a series of three cycles to remove any remaining ammonium bicarbonate. The final dried extracted proteins/peptides were brought up in 20 μl of 0.5% TFA and desalted and concentrated using C_18 _ZipTips (Millipore Corporation, Billerica, MA). Tips were wetted with 10 μl of 100% acetonitrile and equilibrated with 10 μl 0.1% TFA (pH < 4). Samples were then drawn into ZipTip columns by aspirating for 10 cycles and then washed twice with 10 μl 0.1% TFA. Peptides were then eluted from the column twice with 10 μl of 50% acetonitrile, 0.1% TFA. The ZipTip procedure was performed four times for each extract to maximize peptide yield and the elutions were pooled, lyophilized and resuspended in 2 μl of 50% Acetonitrile, 1% TFA for MALDI plate spotting.

Peptides were analyzed by MALDI-ToF/ToF mass spectrometry (5800 Proteomic Analyzer Applied Biosystems, Foster City, CA) in the Mass Spectrometry Core at the Penn State University College of Medicine. All of each ZipTip cleaned samples (1 μl at a time) was applied onto a 384-well MALDI plate (Opti-TOF™ 384 Well Insert, Applied Biosystems) and then 0.7 μl of 2 mg/ml ACH cinnamic acid in 60:40 (acetonitrile: water) was spotted on each well containing peptide. All 13-calibration wells on the MALDI plate were spotted with (1:12 diluted) 4700 calibrant. Autolytic trypsin peptides were also used to internally calibrate the spectra to an accuracy of 20 ppm. Using the GPS Explorer 3.0 software (Applied Biosystems), the MS and MS/MS data were submitted to the MASCOT search engine using the NCBI non-redundant database and mouse taxonomy for identification. The search parameters included: trypsin digestion with a maximum of three missed cleavages; fixed modifications, carbamidomethylation; variable modifications, carbamylation, acetylation, deamidation, oxidation; peptide mass tolerance, 0.15 Da. MASCOT confidence interval scores of > 95% combined with a ProteinPilot score of greater than 61 were considered as a positive protein identification. An image of the reference gel is shown in Figure 1 with all identified proteins circled and numbered. The PANTHER database and the scientific literature were used to provisionally assign molecular function and biological process to each identified protein. We then re-assigned the identified proteins to four broad functional classes including: a) actin-related/cytoskeletal proteins; b) proteins involved in protease balance/chaperone function; c) proteins involved in regulation of inflammation: and d) proteins involved in regulatory/differentiative processes. It should be noted that some proteins are in more than one functional group. This classification scheme was more inclusive than relying solely on the biological function classification provided by PANTHER and similar gene ontology databases. We also used the Ingenuity Pathway Analysis program (Ingenuity Systems, Redwood City, CA) to gain additional insight into the functional significance of the observed changes. Protein names, accession numbers, and the functional groups we assigned them to are listed in Additional File 3 together with a list of supporting references.

### Collection, culture and *in vitro *SP-A treatment of mouse AMs for F-actin staining

AMs were collected from the BAL fluid of male KO mice as described earlier. AMs were washed 1X with AM medium (RPMI-Hepes modification containing 2 mM L-glutamine and 1:100 antibiotic antimycotic solution). AMs were then resuspended in AM medium at 1 × 10^5^/mL and 1 mL (10^5 ^AMs) plated to cover glasses (No. 1 circles, 18 mm, 0.15 mm thick) in 12-well tissue culture plates and allowed to adhere for 90 min at 37°C. The medium was then removed and replaced with 1 mL of fresh AM medium and the cells were cultured overnight at 37°C. AMs were treated with SP-A for either one or six hours by adding 5 μL (5 μg) of a 1 μg/μL human SP-A stock solution in 10 mM Hepes (pH 7.5) directly to the 1 mL of medium from the overnight culture.

### Alexa Fluor 488 phalloidin staining of F-Actin on AMs

After incubation with SP-A, AMs were washed 1X with PBS at 37°C, fixed with 3.7% paraformaldehyde in PBS for 10 min at 37°C and permeabilized with 0.5% Triton in PBS for 5 min. After permeabilization, cells were washed with PBS, incubated for 30 min in staining solution (PBS, 300 nM DAPI, 6.6 nM Alexa Fluor 488 phalloidin) and again washed with PBS 3X for 10 min. Cover glasses containing the AMs were mounted to slides using PBS containing 80% glycerol and 0.3% n-propyl gallate, and then sealed using nail polish.

### Image acquisition and data analysis

AMs were visualized using a Nikon TE-2000 PFS fluorescent microscope with a 60X (1.4 NA) phase contrast lens with a 1.5× tube lens in place and the images captured using a Photometrics Coolsnap HQ2 digital camera (0.07 μm/pixel) and saved as TIFF files. Exposure time (200 ms) was kept constant for all images acquired. Nikon NIS-Elements AR (version 3.0) software was used to do both the image acquisition and data analysis. AMs were analyzed by manually drawing a border for each cell as well as copying an area of equal size to a location immediately adjacent to each cell to be used for background subtraction. Data were then collected for area (cell size), mean fluorescence intensity per pixel of cell area, and the sum of the fluorescence intensity for all of the pixels within a cell. Statistical analysis was performed using Minitab and Excel.

## Competing interests

The authors declare that they have no competing interests.

## Authors' contributions

DSP and JF designed the study. DSP interpreted data and prepared the manuscript. JF assisted with data interpretation, and participated in manuscript preparation. TMU treated mice, collected samples, ran gels, prepared mass spec samples, evaluated mass spec data, did preliminary analysis of proteomic data, and participated in the writing of the manuscript. OAQ prepared the cell samples for the microscopy experiments, collected images, and performed the image analyses. CMY participated in design of actin experiments and interpreted actin data. All authors read and approved the final manuscript.

## Supplementary Material

Additional file 1 **MIAPE: Gel Electrophoresis**. File containing Minimum Information About a Proteomics Experiment-Gel Electrophoresis in the format recommended by the Human Proteome Organization Proteomic Standards Initiative. Click here for file

Additional file 2 **MIAPE: Gel Informatics**. File containing Minimum Information About a Proteomics Experiment-Gel Informatics in the format recommended by the Human Proteome Organization Proteomic Standards Initiative. Click here for file

Additional file 3 **Protein names and cross references to accession numbers and categories**. File containing a table that has the gel numbers and names of all identified proteins. It contains both NCBI GI numbers and Swiss Prot Accession numbers for all proteins, as well as the designation for the functional group(s) to which each protein was assigned. Reference numbers listed in the table are from the reference list in the manuscript. Click here for file

Additional file 4 **Values for all identified alveolar macrophage proteins with note of significant changes**. File containing a table that gives normalized volumes for all proteins for each individual group +/- SD and indicates comparisons between groups that were significantly different. Click here for file

## References

[B1] Hickling TP Malhotra R Bright H McDowell W Blair ED Sim RB Lung surfactant protein A provides a route of entry for respiratory syncytial virus into host cells Viral Immunol 2000 13 125 135 10.1089/vim.2000.13.125 10733174

[B2] Madan T Kishore U Shah A Eggleton P Strong P Wang JY Aggrawal SS Sarma PU Reid KBM Lung surfactant proteins A and D can inhibit specific IgE binding to the allergens of Aspergillus fumigatus and block allergen-induced histamine release from human basophils Clin exp Immunol 1997 110 241 249 9367408 10.1111/j.1365-2249.1997.tb08323.x PMC2265513

[B3] Madan T Kishore U Singh M Strong P Clark H Hussain EM Reid KB Sarma PU Surfactant proteins A and D protect mice against pulmonary hypersensitivity induced by Aspergillus fumigatus antigens and allergens J Clin Invest 2001 107 467 475 10.1172/JCI10124 11181646 PMC199243

[B4] Madan T Reid KB Clark H Singh M Nayak A Sarma PU Hawgood S Kishore U Susceptibility of mice genetically deficient in SP-A or SP-D gene to invasive pulmonary aspergillosis Mol Immunol 2010 47 1923 1930 10.1016/j.molimm.2010.02.027 20413160

[B5] Malhotra R Haurum J Thiel S Jensenius JC Sim RB Pollen grains bind to lung alveolar type II cells (A549) via lung surfactant protein A (SP-A) Biosci Rep 1993 13 79 90 10.1007/BF01145960 8374060

[B6] Kremlev SG Phelps DS Surfactant protein A stimulation of inflammatory cytokine and immunoglobulin production Am J Physiol 1994 267 L712 L719 7810675 10.1152/ajplung.1994.267.6.L712

[B7] Kremlev SG Umstead TM Phelps DS Surfactant protein A regulates cytokine production in the monocytic cell line THP-1 Am J Physiol 1997 272 L996 1004 9176266 10.1152/ajplung.1997.272.5.L996

[B8] Wang G Phelps DS Umstead TM Floros J Human SP-A protein variants derived from one or both genes stimulate TNF-alpha production in the THP-1 cell line Am J Physiol Lung Cell Mol Physiol 2000 278 L946 L954 10781424 10.1152/ajplung.2000.278.5.L946

[B9] Wang H Tracey KJ Gallin JI, Snyderman R Tumor necrosis factor, interleukin-6, macrophage migration inhibitory factor, and macrophage inflammatory protein-1 in inflammation Inflammation: Basic Principles and Clinical Correlates 1999 3 Philadelphia: Lippincott Williams & Wilkins 471 486

[B10] Meloni F Alberti A Bulgheroni A Lupi A Paschetto E Marone BA Rodi G Fietta A Luisetti M Baritussio A Surfactant apoprotein A modulates interleukin-8 and monocyte chemotactic peptide-1 production Eur Respir J 2002 19 1128 1135 10.1183/09031936.02.00211102 12108868

[B11] Borron PJ Crouch EC Lewis JF Wright JR Possmayer F Fraher LJ Recombinant rat surfactant-associated protein D inhibits human T lymphocyte proliferation and IL-2 production J Immunol 1998 161 4599 4603 9794387

[B12] Alcorn JF Wright JR Surfactant protein A inhibits alveolar macrophage cytokine production by CD14-independent pathway Am J Physiol Lung Cell Mol Physiol 2004 286 L129 L136 12959932 10.1152/ajplung.00427.2002

[B13] Gardai SJ Xiao YQ Dickinson M Nick JA Voelker DR Greene KE Henson PM By binding SIRPalpha or calreticulin/CD91, lung collectins act as dual function surveillance molecules to suppress or enhance inflammation Cell 2003 115 13 23 10.1016/S0092-8674(03)00758-X 14531999

[B14] Wang G Umstead TM Phelps DS Al Mondhiry H Floros J The effect of ozone exposure on the ability of human surfactant protein A variants to stimulate cytokine production Environ Health Perspect 2002 110 79 84 11781168 10.1289/ehp.0211079 PMC1240696

[B15] Huang W Wang G Phelps DS Al Mondhiry H Floros J Combined SP-A-bleomycin effect on cytokines by THP-1 cells: impact of surfactant lipids on this effect Am J Physiol Lung Cell Mol Physiol 2002 283 L94 L102 12060565 10.1152/ajplung.00434.2001

[B16] Huang W Wang G Phelps DS Al Mondhiry H Floros J Human SP-A genetic variants and bleomycin-induced cytokine production by THP-1 cells: effect of ozone-induced SP-A oxidation Am J Physiol Lung Cell Mol Physiol 2004 286 L546 L553 10.1152/ajplung.00267.2003 14617519

[B17] Kuronuma K Sano H Kato K Kudo K Hyakushima N Yokota S Takahashi H Fujii N Suzuki H Kodama T Abe S Kuroki Y Pulmonary surfactant protein A augments the phagocytosis of Streptococcus pneumoniae by alveolar macrophages through a casein kinase 2-dependent increase of cell surface localization of scavenger receptor A J Biol Chem 2004 279 21421 21430 10.1074/jbc.M312490200 14993215

[B18] Beharka AA Gaynor CD Kang BK Voelker DR McCormack FX Schlesinger LS Pulmonary surfactant protein A up-regulates activity of the mannose receptor, a pattern recognition receptor expressed on human macrophages J Immunol 2002 169 3565 3573 12244146 10.4049/jimmunol.169.7.3565

[B19] Kremlev SG Phelps DS Effect of SP-A and surfactant lipids on expression of cell surface markers in the THP-1 monocytic cell line Am J Physiol 1997 272 L1070 L1077 9227506 10.1152/ajplung.1997.272.6.L1070

[B20] Gil M McCormack FX LeVine AM Surfactant protein A modulates cell surface expression of CR3 on alveolar macrophages and enhances CR3-mediated phagocytosis J Biol Chem 2009 284 7495 7504 10.1074/jbc.M808643200 19155216 PMC2658045

[B21] Henning LN Azad AK Parsa KV Crowther JE Tridandapani S Schlesinger LS Pulmonary surfactant protein A regulates TLR expression and activity in human macrophages J Immunol 2008 180 7847 7858 18523248 10.4049/jimmunol.180.12.7847 PMC2562757

[B22] Blanco O Catala A Surfactant protein A inhibits the non-enzymatic lipid peroxidation of porcine lung surfactant Prostaglandins Leukot Essent Fatty Acids 2001 65 185 190 10.1054/plef.2001.0309 11728170

[B23] Bridges JP Davis HW Damodarasamy M Kuroki Y Howles G Hui DY McCormack FX Pulmonary surfactant proteins A and D are potent endogenous inhibitors of lipid peroxidation and oxidative cellular injury J Biol Chem 2000 275 38848 38855 10969075 10.1074/jbc.M005322200

[B24] Gil HW Oh MH Woo KM Lee EY Oh MH Hong SY Relationship between pulmonary surfactant protein and lipid peroxidation in lung injury due to paraquat intoxication in rats Korean J Intern Med 2007 22 67 72 10.3904/kjim.2007.22.2.67 17616020 PMC2687609

[B25] Terrasa AM Guajardo MH de Armas SE Catala A Pulmonary surfactant protein A inhibits the lipid peroxidation stimulated by linoleic acid hydroperoxide of rat lung mitochondria and microsomes Biochim Biophys Acta 2005 1735 101 110 15982924 10.1016/j.bbalip.2005.05.007

[B26] Haque R Umstead TM Freeman WM Floros J Phelps DS The impact of surfactant protein-A on ozone-induced changes in the mouse bronchoalveolar lavage proteome Proteome Sci 2009 7 12 10.1186/1477-5956-7-12 19323824 PMC2666657

[B27] Mikerov AN Gan X Umstead TM Miller L Chinchilli VM Phelps DS Floros J Sex differences in the impact of ozone on survival and alveolar macrophage function of mice after Klebsiella pneumoniae infection Respir Res 2008 9 24 10.1186/1465-9921-9-24 18307797 PMC2268931

[B28] Mikerov AN Haque R Gan X Guo X Phelps DS Floros J Ablation of SP-A has a negative impact on the susceptibility of mice to Klebsiella pneumoniae infection after ozone exposure: sex differences Respir Res 2008 9 77 10.1186/1465-9921-9-77 19055785 PMC2655296

[B29] Mikerov AN Umstead TM Gan X Huang W Guo X Wang G Phelps DS Floros J Impact of ozone exposure on the phagocytic activity of human surfactant protein A (SP-A) and SP-A variants Am J Physiol Lung Cell Mol Physiol 2008 294 L121 L130 17981957 10.1152/ajplung.00288.2007 PMC2964667

[B30] Mikerov AN Umstead TM Huang W Liu W Phelps DS Floros J SP-A1 and SP-A2 variants differentially enhance association of Pseudomonas aeruginosa with rat alveolar macrophages Am J Physiol Lung Cell Mol Physiol 2005 288 L150 L158 15377498 10.1152/ajplung.00135.2004

[B31] Kuzmenko AI Wu H McCormack FX Pulmonary collectins selectively permeabilize model bacterial membranes containing rough lipopolysaccharide Biochemistry 2006 45 2679 2685 10.1021/bi0522652 16489761 PMC3156245

[B32] Kuzmenko AI Wu H Wan S McCormack FX Surfactant protein A is a principal and oxidation-sensitive microbial permeabilizing factor in the alveolar lining fluid J Biol Chem 2005 280 25913 25919 10.1074/jbc.M411344200 15890661

[B33] Hickman-Davis JM Gibbs-Erwin J Lindsey JR Matalon S Role of surfactant protein-a in nitric oxide production and Mycoplasma killing in congenic C57BL/6 mice Am J Respir Cell Mol Biol 2004 30 319 325 12959946 10.1165/rcmb.2003-0246OC

[B34] Brinker KG Garner H Wright JR Surfactant protein A modulates the differentiation of murine bone marrow-derived dendritic cells Am J Physiol Lung Cell Mol Physiol 2003 284 L232 L241 12388334 10.1152/ajplung.00187.2002

[B35] Yang S Milla C Panoskaltsis-Mortari A Ingbar DH Blazar BR Haddad IY Human surfactant protein A suppresses T cell-dependent inflammation and attenuates the manifestations of idiopathic pneumonia syndrome in mice Am J Respir Cell Mol Biol 2001 24 527 536 11350821 10.1165/ajrcmb.24.5.4400

[B36] Atochina EN Beck JM Preston AM Haczku A Tomer Y Scanlon ST Fusaro T Casey J Hawgood S Gow AJ Beers MF Enhanced lung injury and delayed clearance of Pneumocystis carinii in surfactant protein A-deficient mice: attenuation of cytokine responses and reactive oxygen-nitrogen species Infect Immun 2004 72 6002 6011 10.1128/IAI.72.10.6002-6011.2004 15385504 PMC517574

[B37] Hickman-Davis JM Fang FC Nathan C Shepherd VL Voelker DR Wright JR Lung surfactant and reactive oxygen-nitrogen species: antimicrobial activity and host-pathogen interactions Am J Physiol Lung Cell Mol Physiol 2001 281 L517 L523 11504674 10.1152/ajplung.2001.281.3.L517

[B38] LeVine AM Kurak KE Bruno MD Stark JM Whitsett JA Korfhagen TR Surfactant protein-A-deficient mice are susceptible to Pseudomonas aeruginosa infection Am J Respir Cell Mol Biol 1998 19 700 708 9761768 10.1165/ajrcmb.19.4.3254

[B39] LeVine AM Kurak KE Wright JR Watford WT Bruno MD Ross GF Whitsett JA Korfhagen TR Surfactant protein-A binds group B streptococcus enhancing phagocytosis and clearance from lungs of surfactant protein-A-deficient mice Am J Respir Cell Mol Biol 1999 20 279 286 9922219 10.1165/ajrcmb.20.2.3303

[B40] LeVine AM Gwozdz J Stark J Bruno M Whitsett J Korfhagen T Surfactant protein-A enhances respiratory syncytial virus clearance in vivo J Clin Invest 1999 103 1015 1021 10.1172/JCI5849 10194474 PMC408263

[B41] Linke MJ Harris CE Korfhagen TR McCormack FX Ashbaugh AD Steele P Whitsett JA Walzer PD Immunosuppressed surfactant protein A-deficient mice have increased susceptibility to Pneumocystis carinii infection J Infect Dis 2001 183 943 952 10.1086/319252 11237812

[B42] Ali M Umstead TM Haque R Mikerov AN Freeman WM Floros J Phelps DS Differences in the BAL proteome after Klebsiella pneumoniae infection in wild type and SP-A-/- mice Proteome Sci 2010 8 34 10.1186/1477-5956-8-34 20565803 PMC2911411

[B43] Guth AM Janssen WJ Bosio CM Crouch EC Henson PM Dow SW Lung environment determines unique phenotype of alveolar macrophages Am J Physiol Lung Cell Mol Physiol 2009 296 L936 L946 10.1152/ajplung.90625.2008 19304907 PMC2692811

[B44] Moulakakis C Stamme C Role of clathrin-mediated endocytosis of surfactant protein A by alveolar macrophages in intracellular signaling Am J Physiol Lung Cell Mol Physiol 2009 296 L430 L441 10.1152/ajplung.90458.2008 19136579

[B45] Breuiller-Fouche M Dubois O Sediki M Garcia-Verdugo I Palaniyar N Tanfin Z Chissey A Cabrol D Charpigny G Mehats C Secreted surfactant protein A from fetal membranes induces stress fibers in cultured human myometrial cells Am J Physiol Endocrinol Metab 2010 298 E1188 E1197 10.1152/ajpendo.00746.2009 20233942

[B46] Umstead TM Freeman WM Chinchilli VM Phelps DS Age-related changes in the expression and oxidation of bronchoalveolar lavage proteins in the rat Am J Physiol Lung Cell Mol Physiol 2009 296 L14 L29 18931054 10.1152/ajplung.90366.2008

[B47] Delanote V Vandekerckhove J Gettemans J Plastins: versatile modulators of actin organization in (patho)physiological cellular processes Acta Pharmacol Sin 2005 26 769 779 10.1111/j.1745-7254.2005.00145.x 15960882

[B48] Wang X Tomso DJ Chorley BN Cho HY Cheung VG Kleeberger SR Bell DA Identification of polymorphic antioxidant response elements in the human genome Hum Mol Genet 2007 16 1188 1200 10.1093/hmg/ddm066 17409198 PMC2805149

[B49] Mullins RD Heuser JA Pollard TD The interaction of Arp2/3 complex with actin: nucleation, high affinity pointed end capping, and formation of branching networks of filaments Proc Natl Acad Sci USA 1998 95 6181 6186 10.1073/pnas.95.11.6181 9600938 PMC27619

[B50] Cooper JA Sept D New insights into mechanism and regulation of actin capping protein Int Rev Cell Mol Biol 2008 267 183 206 18544499 10.1016/S1937-6448(08)00604-7 PMC2583073

[B51] Setiyono A Budiyati AD Purwantomo S Anggelia MR Fanany I Wibowo GA Bachtiar I Utama A Tai S Immunoregulatory effects of AFP domains on monocyte-derived dendritic cell function BMC Immunol 2011 12 4 21235824 10.1186/1471-2172-12-4 PMC3027196

[B52] Morel E Parton RG Gruenberg J Annexin A2-dependent polymerization of actin mediates endosome biogenesis Dev Cell 2009 16 445 457 10.1016/j.devcel.2009.01.007 19289089

[B53] Rescher U Ludwig C Konietzko V Kharitonenkov A Gerke V Tyrosine phosphorylation of annexin A2 regulates Rho-mediated actin rearrangement and cell adhesion J Cell Sci 2008 121 2177 2185 10.1242/jcs.028415 18565825

[B54] Laumonnier Y Syrovets T Burysek L Simmet T Identification of the annexin A2 heterotetramer as a receptor for the plasmin-induced signaling in human peripheral monocytes Blood 2006 107 3342 3349 10.1182/blood-2005-07-2840 16373665

[B55] Jeon YJ Kim DH Jung H Chung SJ Chi SW Cho S Lee SC Park BC Park SG Bae KH Annexin A4 interacts with the NF-kappaB p50 subunit and modulates NF-kappaB transcriptional activity in a Ca2+-dependent manner Cell Mol Life Sci 2010 67 2271 2281 10.1007/s00018-010-0331-9 20237821 PMC11115496

[B56] Potter DA Tirnauer JS Janssen R Croall DE Hughes CN Fiacco KA Mier JW Maki M Herman IM Calpain regulates actin remodeling during cell spreading J Cell Biol 1998 141 647 662 10.1083/jcb.141.3.647 9566966 PMC2132736

[B57] Franco SJ Huttenlocher A Regulating cell migration: calpains make the cut J Cell Sci 2005 118 3829 3838 10.1242/jcs.02562 16129881

[B58] Kostyukova AS Capping complex formation at the slow-growing end of the actin filament Biochemistry (Mosc) 2008 73 1467 1472 10.1134/S0006297908130075 19216712

[B59] Bewley MA Marriott HM Tulone C Francis SE Mitchell TJ Read RC Chain B Kroemer G Whyte MK Dockrell DH A cardinal role for cathepsin d in co-ordinating the host-mediated apoptosis of macrophages and killing of pneumococci PLoS Pathog 2011 7 e1001262 10.1371/journal.ppat.1001262 21298030 PMC3029254

[B60] Kitteringham NR Abdullah A Walsh J Randle L Jenkins RE Sison R Goldring CE Powell H Sanderson C Williams S Higgins L Yamamoto M Hayes J Park BK Proteomic analysis of Nrf2 deficient transgenic mice reveals cellular defence and lipid metabolism as primary Nrf2-dependent pathways in the liver J Proteomics 2010 73 1612 1631 10.1016/j.jprot.2010.03.018 20399915 PMC2891861

[B61] Lundin VF Leroux MR Stirling PC Quality control of cytoskeletal proteins and human disease Trends Biochem Sci 2010 35 288 297 10.1016/j.tibs.2009.12.007 20116259

[B62] Brackley KI Grantham J Subunits of the chaperonin CCT interact with F-actin and influence cell shape and cytoskeletal assembly Exp Cell Res 2010 316 543 553 10.1016/j.yexcr.2009.11.003 19913534

[B63] Lee CG Da Silva CA Dela Cruz CS Ahangari F Ma B Kang MJ He CH Takyar S Elias JA Role of chitin and chitinase/chitinase-like proteins in inflammation, tissue remodeling, and injury Annu Rev Physiol 2011 73 479 501 10.1146/annurev-physiol-012110-142250 21054166 PMC3864643

[B64] Eismann T Huber N Shin T Kuboki S Galloway E Wyder M Edwards MJ Greis KD Shertzer HG Fisher AB Lentsch AB Peroxiredoxin-6 protects against mitochondrial dysfunction and liver injury during ischemia-reperfusion in mice Am J Physiol Gastrointest Liver Physiol 2009 296 G266 G274 19033532 10.1152/ajpgi.90583.2008 PMC2643922

[B65] Singh H Cousin MA Ashley RH Functional reconstitution of mammalian 'chloride intracellular channels' CLIC1, CLIC4 and CLIC5 reveals differential regulation by cytoskeletal actin FEBS J 2007 274 6306 6316 18028448 10.1111/j.1742-4658.2007.06145.x

[B66] Averaimo S Milton RH Duchen MR Mazzanti M Chloride intracellular channel 1 (CLIC1): Sensor and effector during oxidative stress FEBS Lett 2010 584 2076 2084 10.1016/j.febslet.2010.02.073 20385134

[B67] Chuang JZ Chou SY Sung CH Chloride intracellular channel 4 is critical for the epithelial morphogenesis of RPE cells and retinal attachment Mol Biol Cell 2010 21 3017 3028 10.1091/mbc.E09-10-0907 20610659 PMC2929995

[B68] Hu R Xu C Shen G Jain MR Khor TO Gopalkrishnan A Lin W Reddy B Chan JY Kong AN Identification of Nrf2-regulated genes induced by chemopreventive isothiocyanate PEITC by oligonucleotide microarray Life Sci 2006 79 1944 1955 10.1016/j.lfs.2006.06.019 16828809

[B69] Cuzzocrea S Genovese T Failla M Vecchio G Fruciano M Mazzon E Di Paola R Muia C La Rosa C Crimi N Rizzarelli E Vancheri C Protective effect of orally administered carnosine on bleomycin-induced lung injury Am J Physiol Lung Cell Mol Physiol 2007 292 L1095 L1104 10.1152/ajplung.00283.2006 17220373

[B70] Esser J Rakonjac M Hofmann B Fischer L Provost P Schneider G Steinhilber D Samuelsson B Radmark O Coactosin-like protein functions as a stabilizing chaperone for 5-lipoxygenase: role of tryptophan 102 Biochem J 2010 425 265 274 10.1042/BJ20090856 19807693

[B71] Dai H Huang W Xu J Yao B Xiong S Ding H Tang Y Liu H Wu J Shi Y Binding model of human coactosin-like protein with filament actin revealed by mutagenesis Biochim Biophys Acta 2006 1764 1688 1700 17070122 10.1016/j.bbapap.2006.06.017

[B72] Keller A Peltzer J Carpentier G Horvath I Olah J Duchesnay A Orosz F Ovadi J Interactions of enolase isoforms with tubulin and microtubules during myogenesis Biochim Biophys Acta 2007 1770 919 926 10.1016/j.bbagen.2007.01.015 17368730

[B73] Wygrecka M Marsh LM Morty RE Henneke I Guenther A Lohmeyer J Markart P Preissner KT Enolase-1 promotes plasminogen-mediated recruitment of monocytes to the acutely inflamed lung Blood 2009 113 5588 5598 10.1182/blood-2008-08-170837 19182206

[B74] Zanelli CF Valentini SR Is there a role for eIF5A in translation? Amino Acids 2007 33 351 358 10.1007/s00726-007-0533-0 17578650

[B75] Olson EN Nordheim A Linking actin dynamics and gene transcription to drive cellular motile functions Nat Rev Mol Cell Biol 2010 11 353 365 10.1038/nrm2890 20414257 PMC3073350

[B76] Kwak MK Wakabayashi N Itoh K Motohashi H Yamamoto M Kensler TW Modulation of gene expression by cancer chemopreventive dithiolethiones through the Keap1-Nrf2 pathway. Identification of novel gene clusters for cell survival J Biol Chem 2003 278 8135 8145 10.1074/jbc.M211898200 12506115

[B77] Lee JM Calkins MJ Chan K Kan YW Johnson JA Identification of the NF-E2-related factor-2-dependent genes conferring protection against oxidative stress in primary cortical astrocytes using oligonucleotide microarray analysis J Biol Chem 2003 278 12029 12038 10.1074/jbc.M211558200 12556532

[B78] Fernandez JR Byrne B Firestein BL Phylogenetic analysis and molecular evolution of guanine deaminases: from guanine to dendrites J Mol Evol 2009 68 227 235 10.1007/s00239-009-9205-x 19221682

[B79] Kampinga HH Hageman J Vos MJ Kubota H Tanguay RM Bruford EA Cheetham ME Chen B Hightower LE Guidelines for the nomenclature of the human heat shock proteins Cell Stress Chaperones 2009 14 105 111 10.1007/s12192-008-0068-7 18663603 PMC2673902

[B80] Niture SK Jaiswal AK Hsp90 interaction with INrf2(Keap1) mediates stress-induced Nrf2 activation J Biol Chem 2010 285 36865 36875 10.1074/jbc.M110.175802 20864537 PMC2978616

[B81] Wisniewska M Karlberg T Lehtio L Johansson I Kotenyova T Moche M Schuler H Crystal structures of the ATPase domains of four human Hsp70 isoforms: HSPA1L/Hsp70-hom, HSPA2/Hsp70-2, HSPA6/Hsp70B', and HSPA5/BiP/GRP78 PLoS One 2010 5 e8625 10.1371/journal.pone.0008625 20072699 PMC2803158

[B82] Pockley AG Muthana M Calderwood SK The dual immunoregulatory roles of stress proteins Trends Biochem Sci 2008 33 71 79 10.1016/j.tibs.2007.10.005 18182297

[B83] Madore AM Perron S Turmel V Laviolette M Bissonnette EY Laprise C Alveolar macrophages in allergic asthma: An expression signature characterized by heat shock protein pathways Hum Immunol 2009 10.1016/j.humimm.2009.11.005 PMC7124256 19913588

[B84] Thomas SG Calaminus SD Auger JM Watson SP Machesky LM Studies on the actin-binding protein HS1 in platelets BMC Cell Biol 2007 8 46 10.1186/1471-2121-8-46 17996076 PMC2203996

[B85] Burkhardt JK Carrizosa E Shaffer MH The actin cytoskeleton in T cell activation Annu Rev Immunol 2008 26 233 259 10.1146/annurev.immunol.26.021607.090347 18304005

[B86] Gao JL Guillabert A Hu J Le Y Urizar E Seligman E Fang KJ Yuan X Imbault V Communi D Wang JM Parmentier M Murphy PM Migeotte I F2L, a peptide derived from heme-binding protein, chemoattracts mouse neutrophils by specifically activating Fpr2, the low-affinity N-formylpeptide receptor J Immunol 2007 178 1450 1456 17237393 10.4049/jimmunol.178.3.1450

[B87] Li H Liu J Identification of heterogeneous nuclear ribonucleoprotein K as a transactivator for human low density lipoprotein receptor gene transcription J Biol Chem 2010 285 17789 17797 10.1074/jbc.M109.082057 20371611 PMC2878543

[B88] Tsan MF Heat shock proteins and high mobility group box 1 protein lack cytokine function J Leukoc Biol 2011 10.1189/jlb.0810471 21199932

[B89] Goh ET Pardo OE Michael N Niewiarowski A Totty N Volkova D Tsaneva IR Seckl MJ Gout I Involvement of heterogeneous ribonucleoprotein F in the regulation of cell proliferation via the mammalian target of rapamycin/S6 kinase 2 pathway J Biol Chem 2010 285 17065 17076 10.1074/jbc.M109.078782 20308064 PMC2878046

[B90] Yang Y Gozen O Watkins A Lorenzini I Lepore A Gao Y Vidensky S Brennan J Poulsen D Won PJ Li JN Robinson MB Rothstein JD Presynaptic regulation of astroglial excitatory neurotransmitter transporter GLT1 Neuron 2009 61 880 894 10.1016/j.neuron.2009.02.010 19323997 PMC2743171

[B91] Caulin C Ware CF Magin TM Oshima RG Keratin-dependent, epithelial resistance to tumor necrosis factor-induced apoptosis J Cell Biol 2000 149 17 22 10.1083/jcb.149.1.17 10747083 PMC2175089

[B92] Traweek ST Liu J Battifora H Keratin gene expression in non-epithelial tissues. Detection with polymerase chain reaction Am J Pathol 1993 142 1111 1118 7682761 PMC1886881

[B93] Waseem A Alam Y Dogan B White KN Leigh IM Waseem NH Isolation, sequence and expression of the gene encoding human keratin 13 Gene 1998 215 269 279 10.1016/S0378-1119(98)00297-2 9714826

[B94] Herrmann C Golkaramnay E Inman E Rome L Volknandt W Recombinant major vault protein is targeted to neuritic tips of PC12 cells J Cell Biol 1999 144 1163 1172 10.1083/jcb.144.6.1163 10087261 PMC2150576

[B95] Honnappa S Gouveia SM Weisbrich A Damberger FF Bhavesh NS Jawhari H Grigoriev I van Rijssel FJ Buey RM Lawera A Jelesarov I Winkler FK Wuthrich K Akhmanova A Steinmetz MO An EB1-binding motif acts as a microtubule tip localization signal Cell 2009 138 366 376 10.1016/j.cell.2009.04.065 19632184

[B96] Hsu CY Yung BY Down-regulation of nucleophosmin/B23 during retinoic acid-induced differentiation of human promyelocytic leukemia HL-60 cells Oncogene 1998 16 915 923 10.1038/sj.onc.1201615 9484783

[B97] Watanabe N Iwamura T Shinoda T Fujita T Regulation of NFKB1 proteins by the candidate oncoprotein BCL-3: generation of NF-kappaB homodimers from the cytoplasmic pool of p50-p105 and nuclear translocation EMBO J 1997 16 3609 3620 10.1093/emboj/16.12.3609 9218802 PMC1169985

[B98] Wang J Jiao H Stewart TL Shankowsky HA Scott PG Tredget EE Increased severity of bleomycin-induced skin fibrosis in mice with leukocyte-specific protein 1 deficiency J Invest Dermatol 2008 128 2767 2776 10.1038/jid.2008.164 18580965

[B99] Li W Febbraio M Reddy SP Yu DY Yamamoto M Silverstein RL CD36 participates in a signaling pathway that regulates ROS formation in murine VSMCs J Clin Invest 2010 120 3996 4006 10.1172/JCI42823 20978343 PMC2964976

[B100] Natarajan R Salloum FN Fisher BJ Smithson L Almenara J Fowler AA III Prolyl hydroxylase inhibition attenuates post-ischemic cardiac injury via induction of endoplasmic reticulum stress genes Vascul Pharmacol 2009 51 110 118 10.1016/j.vph.2009.05.007 19524066

[B101] Ito Y Kondo E Demachi-Okamura A Akatsuka Y Tsujimura K Tanimoto M Morishima Y Takahashi T Kuzushima K Three immunoproteasome-associated subunits cooperatively generate a cytotoxic T-lymphocyte epitope of Epstein-Barr virus LMP2A by overcoming specific structures resistant to epitope liberation J Virol 2006 80 883 890 10.1128/JVI.80.2.883-890.2006 16378990 PMC1346843

[B102] Hopitzan A Himmelbauer H Spevak W Castanon MJ The mouse Psma1 gene coding for the alpha-type C2 proteasome subunit: structural and functional analysis, mapping, and colocalization with Pde3b on mouse chromosome 7 Genomics 2000 66 313 323 10.1006/geno.2000.6217 10873386

[B103] Jung KA Kwak MK The Nrf2 system as a potential target for the development of indirect antioxidants Molecules 2010 15 7266 7291 10.3390/molecules15107266 20966874 PMC6259123

[B104] Akama K Horikoshi T Sugiyama A Nakahata S Akitsu A Niwa N Intoh A Kakui Y Sugaya M Takei K Imaizumi N Sato T Matsumoto R Iwahashi H Kashiwabara S Baba T Nakamura M Toda T Protein disulfide isomerase-P5, down-regulated in the final stage of boar epididymal sperm maturation, catalyzes disulfide formation to inhibit protein function in oxidative refolding of reduced denatured lysozyme Biochim Biophys Acta 2010 1804 1272 1284 20152940 10.1016/j.bbapap.2010.02.004

[B105] Grune T Reinheckel T Li R North JA Davies KJ Proteasome-dependent turnover of protein disulfide isomerase in oxidatively stressed cells Arch Biochem Biophys 2002 397 407 413 10.1006/abbi.2001.2719 11795901

[B106] Parsyan A Svitkin Y Shahbazian D Gkogkas C Lasko P Merrick WC Sonenberg N mRNA helicases: the tacticians of translational control Nat Rev Mol Cell Biol 2011 12 235 245 10.1038/nrm3083 21427765

[B107] Shih AY Johnson DA Wong G Kraft AD Jiang L Erb H Johnson JA Murphy TH Coordinate regulation of glutathione biosynthesis and release by Nrf2-expressing glia potently protects neurons from oxidative stress J Neurosci 2003 23 3394 3406 12716947 10.1523/JNEUROSCI.23-08-03394.2003 PMC6742304

[B108] Liu P Bartz R Zehmer JK Ying YS Zhu M Serrero G Anderson RG Rab-regulated interaction of early endosomes with lipid droplets Biochim Biophys Acta 2007 1773 784 793 10.1016/j.bbamcr.2007.02.004 17395284 PMC2676670

[B109] Rivero F Illenberger D Somesh BP Dislich H Adam N Meyer AK Defects in cytokinesis, actin reorganization and the contractile vacuole in cells deficient in RhoGDI EMBO J 2002 21 4539 4549 10.1093/emboj/cdf449 12198156 PMC126189

[B110] Benarafa C Cooley J Zeng W Bird PI Remold-O'Donnell E Characterization of four murine homologs of the human ov-serpin monocyte neutrophil elastase inhibitor MNEI (SERPINB1) J Biol Chem 2002 277 42028 42033 10.1074/jbc.M207080200 12189154

[B111] Fang L Min L Lin Y Ping G Rui W Ying Z Xi W Ting H Li L Ke D Jihong R Huizhong Z Downregulation of stathmin expression is mediated directly by Egr1 and associated with p53 activity in lung cancer cell line A549 Cell Signal 2010 22 166 173 10.1016/j.cellsig.2009.09.030 19786090

[B112] Eng EW Bettio A Ibrahim J Harrison RE MTOC reorientation occurs during FcgammaR-mediated phagocytosis in macrophages Mol Biol Cell 2007 18 2389 2399 10.1091/mbc.E06-12-1128 17442887 PMC1924806

[B113] Zuo S Xue Y Tang S Yao J Du R Yang P Chen X 14-3-3 epsilon dynamically interacts with key components of mitogen-activated protein kinase signal module for selective modulation of the TNF-alpha-induced time course-dependent NF-kappaB activity J Proteome Res 2010 9 3465 3478 10.1021/pr9011377 20462248

[B114] Bodas M Min T Vij N Early-age-related changes in proteostasis augment immunopathogenesis of sepsis and acute lung injury PLoS One 2010 5 e15480 10.1371/journal.pone.0015480 21085581 PMC2981560

[B115] Mor-Vaknin N Punturieri A Sitwala K Markovitz DM Vimentin is secreted by activated macrophages Nat Cell Biol 2003 5 59 63 10.1038/ncb898 12483219

[B116] Varin A Gordon S Alternative activation of macrophages: immune function and cellular biology Immunobiol 2009 214 630 641 10.1016/j.imbio.2008.11.009 19264378

[B117] Jin M Opalek JM Marsh CB Wu HM Proteome comparison of alveolar macrophages with monocytes reveals distinct protein characteristics Am J Respir Cell Mol Biol 2004 31 322 329 10.1165/rcmb.2004-0080OC 15130903

[B118] Mosser DM Edwards JP Exploring the full spectrum of macrophage activation Nat Rev Immunol 2008 8 958 969 10.1038/nri2448 19029990 PMC2724991

[B119] Kang MI Kobayashi A Wakabayashi N Kim SG Yamamoto M Scaffolding of Keap1 to the actin cytoskeleton controls the function of Nrf2 as key regulator of cytoprotective phase 2 genes Proc Natl Acad Sci USA 2004 101 2046 2051 10.1073/pnas.0308347100 14764898 PMC357049

[B120] Goto H Ledford JG Mukherjee S Noble PW Williams KL Wright JR The role of surfactant protein A in bleomycin-induced acute lung injury Am J Respir Crit Care Med 2010 181 1336 1344 10.1164/rccm.200907-1002OC 20167853 PMC2894409

[B121] Wang G Guo X DiAngelo S Thomas NJ Floros J Humanized SFTPA1 and SFTPA2 transgenic mice reveal functional divergence of SP-A1 and SP-A2: formation of tubular myelin in vivo requires both gene products J Biol Chem 2010 285 11998 12010 10.1074/jbc.M109.046243 20048345 PMC2852938

[B122] Chabot S Salez L McCormack FX Touqui L Chignard M Surfactant protein A inhibits lipopolysaccharide-induced in vivo production of interleukin-10 by mononuclear phagocytes during lung inflammation Am J Respir Cell Mol Biol 2003 28 347 353 10.1165/rcmb.4883 12594061

[B123] Linke MJ Ashbaugh AA Koch JV Levin L Tanaka R Walzer PD Effects of surfactant protein-A on the interaction of Pneumocystis murina with its host at different stages of the infection in mice J Eukaryot Microbiol 2009 56 58 65 10.1111/j.1550-7408.2008.00363.x 19335775 PMC2675919

[B124] Morrow DM Entezari-Zaher T Romashko J Azghani AO Javdan M Ulloa L Miller EJ Mantell LL Antioxidants preserve macrophage phagocytosis of Pseudomonas aeruginosa during hyperoxia Free Radic Biol Med 2007 42 1338 1349 10.1016/j.freeradbiomed.2007.01.031 17395007 PMC3104269

[B125] Ramadas RA Wu L LeVine AM Surfactant protein A enhances production of secretory leukoprotease inhibitor and protects it from cleavage by matrix metalloproteinases J Immunol 2009 182 1560 1567 19155504 10.4049/jimmunol.182.3.1560

[B126] Vazquez de Lara LG Umstead TM Davis SE Phelps DS Surfactant protein A increases matrix metalloproteinase-9 production by THP-1 cells Am J Physiol Lung Cell Mol Physiol 2003 285 L899 L906 12842807 10.1152/ajplung.00082.2003

[B127] Guillot L Balloy V McCormack FX Golenbock DT Chignard M Si-Tahar M Cutting edge: the immunostimulatory activity of the lung surfactant protein-A involves Toll-like receptor 4 J Immunol 2002 168 5989 5992 12055204 10.4049/jimmunol.168.12.5989

[B128] Murakami S Iwaki D Mitsuzawa H Sano H Takahashi H Voelker DR Akino T Kuroki Y Surfactant protein A inhibits peptidoglycan-induced tumor necrosis factor-alpha secretion in U937 cells and alveolar macrophages by direct interaction with toll-like receptor 2 J Biol Chem 2002 277 6830 6837 10.1074/jbc.M106671200 11724772

[B129] Park J Choe SS Choi AH Kim KH Yoon MJ Suganami T Ogawa Y Kim JB Increase in glucose-6-phosphate dehydrogenase in adipocytes stimulates oxidative stress and inflammatory signals Diabetes 2006 55 2939 2949 10.2337/db05-1570 17065329

[B130] LeVine AM Hartshorn K Elliott J Whitsett J Korfhagen T Absence of SP-A modulates innate and adaptive defense responses to pulmonary influenza infection Am J Physiol Lung Cell Mol Physiol 2002 282 L563 L572 11839553 10.1152/ajplung.00280.2001

[B131] Famuyide ME Hasday JD Carter HC Chesko KL He JR Viscardi RM Surfactant protein-A limits Ureaplasma-mediated lung inflammation in a murine pneumonia model Pediatr Res 2009 66 162 167 10.1203/PDR.0b013e3181aabd66 19390477 PMC2758107

[B132] Yang S Milla C Panoskaltsis-Mortari A Hawgood S Blazar BR Haddad IY Surfactant protein A decreases lung injury and mortality after murine marrow transplantation Am J Respir Cell Mol Biol 2002 27 297 305 12204891 10.1165/rcmb.2002-0035OC

[B133] Ishii T Itoh K Takahashi S Sato H Yanagawa T Katoh Y Bannai S Yamamoto M Transcription factor Nrf2 coordinately regulates a group of oxidative stress-inducible genes in macrophages J Biol Chem 2000 275 16023 16029 10.1074/jbc.275.21.16023 10821856

[B134] Kustermans G El Mjiyad N Horion J Jacobs N Piette J Legrand-Poels S Actin cytoskeleton differentially modulates NF-kappaB-mediated IL-8 expression in myelomonocytic cells Biochem Pharmacol 2008 76 1214 1228 10.1016/j.bcp.2008.08.017 18789311

[B135] Koptides M Umstead TM Floros J Phelps DS Surfactant protein A activates NF-kappa B in the THP-1 monocytic cell line Am J Physiol 1997 273 L382 L388 9277450 10.1152/ajplung.1997.273.2.L382

[B136] Song M Phelps DS Interaction of surfactant protein A with lipopolysaccharide and regulation of inflammatory cytokines in the THP-1 monocytic cell line Infect Immun 2000 68 6611 6617 10.1128/IAI.68.12.6611-6617.2000 11083772 PMC97757

[B137] Nair S Doh ST Chan JY Kong AN Cai L Regulatory potential for concerted modulation of Nrf2- and Nfkb1-mediated gene expression in inflammation and carcinogenesis Br J Cancer 2008 99 2070 2082 10.1038/sj.bjc.6604703 19050705 PMC2607222

[B138] Liu GH Qu J Shen X NF-kappaB/p65 antagonizes Nrf2-ARE pathway by depriving CBP from Nrf2 and facilitating recruitment of HDAC3 to MafK Biochim Biophys Acta 2008 1783 713 727 10.1016/j.bbamcr.2008.01.002 18241676

[B139] Wu MC Lin X Prior biological knowledge-based approaches for the analysis of genome-wide expression profiles using gene sets and pathways Stat Methods Med Res 2009 18 577 593 10.1177/0962280209351925 20048386 PMC2827341

[B140] Goeman JJ Buhlmann P Analyzing gene expression data in terms of gene sets: methodological issues Bioinformatics 2007 23 980 987 10.1093/bioinformatics/btm051 17303618

[B141] Floros J Phelps DS Nakos G, Lekka M Pulmonary surfactant protein A; structure, expression, and its role in innate host defense Update of intensive care medicine 2002 6 Ioannina Greece: University of Ioannina 87 102

[B142] Haque R Umstead TM Ponnuru P Guo X Hawgood S Phelps DS Floros J Role of surfactant protein-A (SP-A) in lung injury in response to acute ozone exposure of SP-A deficient mice Toxicol Appl Pharmacol 2007 220 72 82 10.1016/j.taap.2006.12.017 17307210 PMC1906716

[B143] Umstead TM Lu CJ Freeman WM Myers JL Clark JB Thomas NJ Chinchilli VM Vrana KE Undar A Phelps DS Dual-platform proteomics study of plasma biomarkers in pediatric patients undergoing cardiopulmonary bypass Pediatr Res 2010 67 641 649 10.1203/PDR.0b013e3181dceef5 20308938

