# Supplementary material for: In vivo rescue of alveolar macrophages from SP-A knockout mice with exogenous SP-A nearly restores a wild type intracellular proteome; actin involvement
Source: Proteome Sci. 2011 Oct 28;9:67. doi: 10.1186/1477-5956-9-67 (PMC3219558; doi:10.1186/1477-5956-9-67)
Supplement: Additional file 3 — Protein names and cross references to accession numbers and categories. File containing a table that has the gel numbers and names of all identified proteins. It contains both NCBI GI numbers and Swiss Prot Accession numbers for all proteins, as well as the designation for the functional group(s) to which each protein was assigned. Reference numbers listed in the table are from the reference list in the manuscript. [file 1477-5956-9-67-S3.DOC]

**Additional File 3**

**Protein names and cross references to accession numbers and categories**.

| **Gel No.** | **Protein Name** | **NCBI**  **GI Number** | **Swiss-Prot**  **Access. No.** | **Functional**  **Categories** | **Refs.** |
| --- | --- | --- | --- | --- | --- |
| 1 | 65-kDa macrophage protein | gi|984636 | Q61233 | ARC,NRF | [47,48] |
| 2 | Actin related protein 2/3 complex, subunit 5 | gi|224809382 | Q9CPW4 | ARC | [49,50] |
| 3 | Actin-related protein 3 | gi|12835802 | Q99JY9 | ARC | [49] |
| 4 | Actr2 protein | gi|29126784 | P61161 | ARC | [49] |
| 5 | Alpha-fetoprotein | gi|191765 | P07724 | ROI | [51] |
| 6 | Annexin A2 | gi|6996913 | P07356 | ARC, ROI | [52-54] |
| 7 | Annexin A4 | gi|33416530 | Q7TMN7 | ROI | [55] |
| 8 | Anxa 5 protein | gi|13277612 | P48036 |  |  |
| 9 | ArsA arsenite transporter, ATP-binding, homolog 1 | gi|12025542 | O54984 |  |  |
| 10 | Atp5b protein | gi|23272966 | P56480 |  |  |
| 11 | Calpain, small subunit 1 | gi|110227381 | O88456 | ARC, PBCF | [56,57] |
| 12 | Capping protein (actin filament) muscle Z-line, alpha 2 (CapZ alpha-2) | gi|6671672 | P47754 | ARC | [50,58] |
| 13 | Capping protein (actin filament) muscle Z-line, beta isoform (CapZ beta) | gi|83649737 | P47757 | ARC | [50,58] |
| 14 | Cathepsin D precursor | gi|6753556 | P18242 | PBCF, NRF | [59,60] |
| 15 | Chaperonin subunit 2 (beta) (CCT2) | gi|126521835 | Q542X7 | ARC,PBCF | [61,62] |
| 16 | Chia protein | gi|15029822 | Q91XA9 | ROI | [63] |
| 17 | Chitinase 3-like 3 precursor (Ym1) | gi|254281348 | O35744 | ROI | [63] |
| 18 | Chitinase-related protein MCRP | gi|1336166 | Q61201 | ROI | [64] |
| 19 | Chloride intracellular channel 1 | gi|15617203 | Q9Z1Q5 | ARC, PBCF | [65,66] |
| 20 | Chloride intracellular channel 4 (mitochondrial) | gi|7304963 | Q9QYB1 | ARC, NRF | [67,68] |
| 21 | CNDP dipeptidase 2 | gi|31981273 | Q9D1A2 | PBCF | [69] |
| 22 | Coactosin-like 1 | gi|19482160 | Q9CQI6 | ARC, PBCF | [70,71] |
| 23 | EF hand domain containing 2 | gi|31981086 | Q8C845 |  |  |
| 24 | Eno1 protein (Alpha-enolase) | gi|34784434 | Q6PHC1 | ARC, PBCF, ROI | [55,72,73] |
| 25 | Eukaryotic translation initiation factor 5A | gi|56800106 | P63242 | ARC, RDP, ROI | [74] |
| 26 | Ezrin | gi|50881 | P26040 | ARC | [56] |
| 27 | F-actin capping protein alpha-1 subunit (CapZ alpha-1) | gi|161086971 | P47753 | ARC | [50,58] |
| 28 | Ferritin heavy chain 1 | gi|6753912 | P09528 | NRF | [48] |
| 29 | Ferritin light chain 1 | gi|114326466 | Q9CPX4 | NRF | [48] |
| 30 | Gamma-actin | gi|809561 | P63260 | ARC, NRF | [75,76] |
| 31 | Gelsolin precursor | gi|28916693 | P13020 | ARC, NRF | [75,76] |
| 32 | Glucose-6-phosphate dehydrogenase X-linked | gi|6996917 | Q00612 | NRF | [77] |
| 33 | Guanine deaminase | gi|6753960 | Q9R111 | ARC | [78] |
| 34 | Heat shock protein 1, beta (HSP90AB1) | gi|40556608 | Q71LX8 | ARC, NRF, PBCF, ROI | [76,79,80] |
| 35 | Heat shock protein 5 precursor (GRP78) | gi|254540166 | P20029 | NRF, PBCF, ROI | [48,79,81,82] |
| 36 | Heat shock protein 65 (HSP60) | gi|51455 | P63038 | PBCF, ROI | [79,82,83] |
| 37 | Heat shock protein 8 (HSC70; HSC71) | gi|42542422 | P63017 | PBCF, ROI | [79,82] |
| 38 | Heat shock protein 90, beta (Grp94), member 1 | gi|14714615 | Q91V38 | NRF, PBCF, ROI | [79,82] |
| 39 | Hematopoietic cell specific Lyn substrate 1 | gi|255760028 | Q922I8 | ARC, ROI | [84,85] |
| 40 | Heme-binding protein | gi|3724328 | Q9R257 | ROI | [86] |
| 41 | Heterogeneous nuclear ribonucleoprotein K | gi|13384620 | P61979 | RDP | [87] |
| 42 | High mobility group 1 protein | gi|600761 | P63158 | RDP, ROI | [88] |
| 43 | Hnrpf protein | gi|58476100 | Q9Z2X1 | RDP | [89] |
| 44 | Kappa-B motif-binding phosphoprotein | gi|1083569 | Q9YH06 | RDP | [90] |
| 45 | Keratin complex 2, basic, gene 8 | gi|114145561 | P11679 | ARC, NRF | [48,91,92] |
| 46 | Keratin type II | gi|511654 | P11679 | ARC | [92] |
| 47 | Krt13 protein | gi|37994713 | P08730 | ARC, NRF | [48,92,93] |
| 48 | Laminin receptor | gi|293694 | P14206 |  |  |
| 49 | Major vault protein (MVP) | gi|17433104 | Q9EQK5 | ARC | [94] |
| 50 | Microtubule-associated protein, RP/EB family, member 1 | gi|7106301 | Q61166 | ARC | [95] |
| 51 | Myosin light chain, regulatory B-like | gi|71037403 | Q6ZWQ9 | ARC | [75] |
| 52 | Nucleophosmin 1 | gi|55153941 | Q5U438 | RDP | [96] |
| 53 | p50b, Leukocyte-specific protein 1 (LSP1) | gi|728498 | P19973 | ARC, RDP, ROI | [97,98] |
| 54 | Peroxiredoxin 2 | gi|148747558 | Q61171 | NRF | [99] |
| 55 | Prolyl 4-hydroxylase, beta polypeptide precursor | gi|42415475 | Q922C8 | PBCF | [100] |
| 56 | Proteasome (prosome, macropain) 28 subunit, alpha | gi|6755212 | P97371 | PBCF | [101] |
| 57 | Proteasome alpha 1 subunit | gi|33563282 | Q9R1P4 | PBCF, NRF | [102,103] |
| 58 | Protein disulfide isomerase associated 6 (PDI-P5) | gi|60502437 | Q922R8 | PBCF | [104] |
| 59 | Protein disulfide-isomerase A3 precursor | gi|112293264 | P27773 | PBCF | [105] |
| 60 | Protein synthesis initiation factor 4A | gi|556308 | P60843 | NRF, RDP | [48,106] |
| 61 | Purine nucleoside phosphorylase | gi|388921 | P23492 |  |  |
| 62 | Put. beta-actin (aa 27-375) | gi|49868 | P60710 | ARC, NRF | [75,107] |
| 63 | Rab GDP dissociation inhibitor beta | gi|26348171 | Q61598 | ARC | [108] |
| 64 | Rho GDP dissociation inhibitor (GDI) alpha | gi|31982030 | Q99PT1 | ARC | [109] |
| 65 | Rho, GDP dissociation inhibitor (GDI) beta | gi|33563236 | Q61599 | ARC | [109] |
| 66 | Serine (or cysteine) proteinase inhibitor, clade B, member 1a | gi|114158675 | Q9D154 | PBCF | [110] |
| 67 | Stathmin | gi|14625464 | Q91XT3 | ARC | [111] |
| 68 | Superoxide dismutase 1, soluble | gi|45597447 | P08228 | NRF | [48] |
| 69 | Tropomodulin 3 | gi|8394460 | Q9JHJ0 | ARC | [58] |
| 70 | Tropomyosin 3, gamma | gi|40254525 | Q8K0Z5 | ARC | [58] |
| 71 | Tubulin, beta 5 | gi|7106439 | P99024 | ARC, NRF | [76,112] |
| 72 | Tyrosine 3/tryptophan 5 -monooxygenase activation protein, ε (14-3-3ε) | gi|5803225 | P62258 | ROI | [113] |
| 73 | Tyrosine 3-monooxyg./tryptophan 5-monooxyg. activation protein, β (14-3-3β) | gi|31543974 | Q9CQV8 | ROI | [113] |
| 74 | Vacuolar adenosine triphosphatase subunit B | gi|1184661 | P62814 |  |  |
| 75 | Valosin-containing protein | gi|6005942 | P55072 | ARC, NRF, PBCF | [48,114] |
| 76 | Vimentin | gi|31982755 | P20152 | ARC, NRF, ROI | [48,115] |

List of proteins identified by 2D-DIGE by gel number with cross references to NCBI GI number, Swiss-Prot accession number, and functional protein categories (ARC, actin-related/cytoskeletal; NRF, Nrf2-regulated proteins; PBCF, protease balance/chaperone function; ROI, regulation of inflammation; and RDP, regulatory/ differentiative processes). Classification into Functional Categories was based on information in the references cited (see reference list in manuscript).
