# Supplementary material for: In vivo rescue of alveolar macrophages from SP-A knockout mice with exogenous SP-A nearly restores a wild type intracellular proteome; actin involvement
Source: Proteome Sci. 2011 Oct 28;9:67. doi: 10.1186/1477-5956-9-67 (PMC3219558; doi:10.1186/1477-5956-9-67)
Supplement: Additional file 4 — Values for all identified alveolar macrophage proteins with note of significant changes. File containing a table that gives normalized volumes for all proteins for each individual group +/- SD and indicates comparisons between groups that were significantly different. [file 1477-5956-9-67-S4.DOC]

**Additional File 4**

**Values for all identified alveolar macrophage proteins with note of significant changes**.

| **Gel No.** | **Protein Name** | **KO**  **[Group 1]** | **KO 6hr SP-A**  **[Group 2]** | **KO 18 hr SP-A**  **[Group 3]** | **WT**  **[Group 4]** |
| --- | --- | --- | --- | --- | --- |
| 1 | 65-kDa macrophage protein | 6.29 (0.383)^3^ | 5.86 (0.326) | 5.48 (0.405)^1,4^ | 6.12 (0.308)^3^ |
| 2 | Actin related protein 2/3 complex, subunit 5 | 0.96 (0.064) | 0.95 (0.077) | 0.96 (0.059) | 1.00 (0.034) |
| 3 | Actin-related protein 3 | 2.00 (0.032)^2,3^ | 1.92 (0.049)^1^ | 1.86 (0.061)^1,4^ | 1.95 (0.032)^3^ |
| 4 | Actr2 protein | 1.01 (0.072) | 0.93 (0.045) | 0.96 (0.067) | 0.96 (0.073) |
| 5 | Alpha-fetoprotein | 7.34 (0.189)^2,3^ | 10.82 (1.382)^1,4^ | 9.19 (1.391)^1^ | 7.39 (0.731)^2^ |
| 6 | Annexin A2 | 2.28 (0.061)^2,3,4^ | 2.03 (0.121)^1^ | 2.09 (0.094)^1^ | 2.11 (0.074)^1^ |
| 7 | Alpha-fetoprotein | 7.34 (0.189) | 10.82 (1.382) | 9.19 (1.391) | 7.39 (0.731) |
| 8 | Annexin A2 | 2.28 (0.061) | 2.03 (0.121) | 2.09 (0.094) | 2.11 (0.074) |
| 9 | ArsA arsenite transporter, ATP-binding, homolog 1 | 1.11 (0.091)^2^ | 0.96 (0.038)^1^ | 1.05 (0.100) | 1.02 (0.049) |
| 10 | Atp5b protein | 2.22 (0.153)^3,4^ | 2.02 (0.169) | 1.89 (0.112)^1^ | 1.91 (0.027)^1^ |
| 11 | Calpain, small subunit 1 | 1.12 (0.064)^3^ | 1.04 (0.097) | 1.01 (0.061)^1^ | 1.08 (0.041) |
| 12 | Capping protein (actin filament) muscle Z-line, alpha 2 (CapZ alpha-2) | 1.09 (0.038) | 1.03 (0.106) | 1.11 (0.051) | 1.20 (0.226) |
| 13 | Capping protein (actin filament) muscle Z-line, beta isoform a (CapZ beta) | 1.14 (0.046)^2^ | 1.03 (0.021)^1^ | 1.04 (0.083) | 1.07 (0.049) |
| 14 | Cathepsin D precursor | 1.09 (0.017) | 1.00 (0.075) | 1.08 (0.098) | 1.07 (0.108) |
| 15 | Chaperonin subunit 2 (beta) (CCT) | 2.18 (0.082)^3,4^ | 2.05 (0.141) | 1.93 (0.098)^1^ | 1.91 (0.119)^1^ |
| 16 | Chia protein | 1.12 (0.181) | 1.03 (0.167) | 1.15 (0.072)^4^ | 0.92 (0.137)^3^ |
| 17 | Chitinase 3-like 3 precursor (Ym1) | 0.88 (0.049)^3,4^ | 1.02 (0.386) | 0.81 (0.011)^1,4^ | 0.73 (0.018)^1,3^ |
| 18 | Chitinase-related protein MCRP | 0.46 (0.045) | 1.09 (1.175) | 0.57 (0.112)^4^ | 0.40 (0.043)^3^ |
| 19 | Chloride intracellular channel 1 | 2.23 (0.115)^3,4^ | 2.12 (0.078) | 2.06 (0.055)^1^ | 2.00 (0.107)^1^ |
| 20 | Chloride intracellular channel 4 (mitochondrial) | 1.72 (0.213)^3^ | 2.15 (0.688) | 2.73 (0.591)^1^ | 2.68 (1.296) |
| 21 | CNDP dipeptidase 2 | 2.08 (0.086)^2,3^ | 1.94 (0.054)^1^ | 1.86 (0.132)^1^ | 2.04 (0.192) |
| 22 | Coactosin-like 1 | 2.39 (0.232)^3^ | 2.99 (1.085) | 3.93 (0.680)^1^ | 4.24 (2.111) |
| 23 | EF hand domain containing 2 | 1.06 (0.062) | 0.98 (0.036)^3,4^ | 1.07 (0.044)^2^ | 1.16 (0.142)^2^ |
| 24 | Eno1 protein (Alpha-enolase) | 1.92 (0.086) | 1.95 (0.328) | 1.89 (0.172) | 1.82 (0.065) |
| 25 | Eukaryotic translation initiation factor 5A | 1.76 (0.256) | 1.79 (0.098) | 1.96 (0.169) | 1.84 (0.119) |
| 26 | Ezrin | 5.33 (0.216)^3,4^ | 4.82 (0.357) | 4.70 (0.318)^1^ | 4.72 (0.110)^1^ |
| 27 | F-actin capping protein alpha-1 subunit (CapZ alpha-1) | 1.97 (0.178) | 2.02 (0.361) | 2.20 (0.229) | 2.30 (0.539) |
| 28 | Ferritin heavy chain 1 | 3.14 (0.046)^3^ | 2.92 (0.207)^3^ | 3.51 (0.133)^1,2,4^ | 2.98 (0.289)^3^ |
| 29 | Ferritin light chain 1 | 2.35 (0.204)^2^ | 2.01 (0.135)^1^ | 2.18 (0.169) | 2.12 (0.099) |
| 30 | Gamma-actin | 18.30 (0.439)^3,4^ | 17.59 (0.400)^3^ | 16.51 (0.562)^1,2^ | 17.08 (0.211)^1^ |
| 31 | Gelsolin precursor | 6.12 (0.254) | 6.79 (0.497)^3^ | 5.92 (0.459)^2^ | 6.23 (0.581) |
| 32 | Glucose-6-phosphate dehydrogenase X-linked | 1.04 (0.027)^3,4^ | 0.99 (0.093) | 0.92 (0.042)^1^ | 0.96 (0.057)^1^ |
| 33 | Guanine deaminase | 1.90 (0.036) | 1.83 (0.095) | 1.87 (0.072) | 1.89 (0.090) |
| 34 | Heat shock protein 1, beta (HSP90AB1) | 0.95 (0.050) | 0.96 (0.090) | 0.88 (0.031) | 0.94 (0.075) |
| 35 | Heat shock protein 5 precursor (GRP78) | 3.87 (0.160)^3^ | 3.91 (0.288) | 3.54 (0.150)^1^ | 3.67 (0.145) |
| 36 | Heat shock protein 65 (HSP60) | 2.14 (0.177)^3,4^ | 2.00 (0.213) | 1.84 (0.102)^1^ | 1.84 (0.059)^1^ |
| 37 | Heat shock protein 8 (HSC70; HSC71) | 3.97 (0.254) | 4.11 (0.243) | 4.12 (0.224) | 3.96 (0.205) |
| 38 | Heat shock protein 90, beta (Grp94), member 1 | 1.04 (0.022)^3,4^ | 0.99 (0.074)^3^ | 0.89 (0.041)^1,2^ | 0.92 (0.048)^1^ |
| 39 | Hematopoietic cell specific Lyn substrate 1 | 3.71 (0.686) | 3.71 (0.687) | 3.89 (0.324) | 4.00 (1.316) |
| 40 | Heme-binding protein | 1.21 (0.176)^4^ | 1.20 (0.063)^4^ | 1.10 (0.217)^4^ | 0.62 (0.059)^1,2,3^ |
| 41 | Heterogeneous nuclear ribonucleoprotein K | 1.21 (0.095)^2,3,4^ | 1.06 (0.077)^1^ | 0.97 (0.063)^1^ | 1.05 (0.037)^1^ |
| 42 | High mobility group 1 protein | 1.00 (0.211) | 1.11 (0.129) | 0.97 (0.195) | 1.03 (0.122) |
| 43 | Hnrpf protein | 2.06 (0.081) | 1.97 (0.045) | 1.93 (0.097) | 2.02 (0.086) |
| 44 | Kappa-B motif-binding phosphoprotein | 3.37 (0.384) | 3.25 (0.302) | 3.14 (0.276) | 3.22 (0.253) |
| 45 | Keratin complex 2, basic, gene 8 | 1.69 (0.078)^4^ | 1.68 (0.237)^4^ | 1.78 (0.159)^4^ | 5.04 (1.708)^1,2,3^ |
| 46 | Keratin type II | 2.21 (0.126)^3^ | 2.01 (0.134) | 1.87 (0.178)^1,4^ | 2.19 (0.081)^3^ |
| 47 | Krt13 protein | 0.57 (0.088)^3^ | 1.12 (1.055) | 0.73 (0.089)^1,4^ | 0.51 (0.079)^3^ |
| 48 | Laminin receptor | 2.02 (0.082) | 1.92 (0.066) | 2.02 (0.150) | 1.96 (0.039) |
| 49 | Major vault protein (MVP) | 3.26 (0.098)^2,3,4^ | 2.98 (0.169)^1^ | 2.99 (0.108)^1,4^ | 2.73 (0.139)^1,3^ |
| 50 | Microtubule-associated protein, RP/EB family, member 1 | 0.96 (0.080)^3^ | 0.95 (0.105) | 0.83 (0.043)^1^ | 0.86 (0.075) |
| 51 | Myosin light chain, regulatory B-like | 4.73 (0.281) | 4.92 (0.823) | 4.88 (0.480) | 4.92 (0.185) |
| 52 | Nucleophosmin 1 | 4.32 (0.346) | 4.12 (0.335) | 3.95 (0.169) | 3.94 (0.273) |
| 53 | p50b | 1.17 (0.040) | 1.10 (0.144) | 1.10 (0.085) | 1.13 (0.060) |
| 54 | Peroxiredoxin 2 | 1.04 (0.126) | 0.98 (0.099) | 1.04 (0.046) | 1.07 (0.037) |
| 55 | Prolyl 4-hydroxylase, beta polypeptide precursor | 3.94 (0.116)^2,4^ | 4.43 (0.364)^1,4^ | 4.01 (0.165)^4^ | 3.40 (0.162)^1,2,3^ |
| 56 | Proteasome (prosome, macropain) 28 subunit, alpha | 1.96 (0.108) | 1.95 (0.238) | 1.99 (0.082) | 2.17 (0.187) |
| 57 | Proteasome alpha 1 subunit | 1.10 (0.083) | 1.04 (0.069) | 1.01 (0.113) | 0.99 (0.048) |
| 58 | Protein disulfide isomerase associated 6 (PDI-P5) | 3.79 (0.267) | 3.57 (0.182) | 3.64 (0.264) | 3.75 (0.370) |
| 59 | Protein disulfide-isomerase A3 precursor | 3.79 (0.264) | 3.76 (0.268) | 3.53 (0.104) | 3.60 (0.132) |
| 60 | Protein synthesis initiation factor 4A | 1.07 (0.083)^4^ | 1.10 (0.167) | 0.99 (0.044)^4^ | 0.91 (0.028)^1,3^ |
| 61 | Purine nucleoside phosphorylase | 1.22 (0.792) | 0.96 (0.336)^4^ | 0.77 (0.118)^4^ | 1.53 (0.226)^2,3^ |
| 62 | Put. beta-actin (aa 27-375) | 3.19 (0.907)^3^ | 4.32 (2.198) | 6.32 (1.434)^1^ | 6.18 (3.248) |
| 63 | Rab GDP dissociation inhibitor beta | 1.08 (0.053)^3,4^ | 1.01 (0.035)^3^ | 0.94 (0.032)^1,2^ | 0.96 (0.042)^1^ |
| 64 | Rho GDP dissociation inhibitor (GDI) alpha | 1.78 (0.111) | 1.93 (0.321) | 1.75 (0.038) | 1.73 (0.024) |
| 65 | Rho, GDP dissociation inhibitor (GDI) beta | 2.12 (0.225) | 2.63 (0.500) | 2.36 (0.240) | 2.24 (0.165) |
| 66 | Serine (or cysteine) proteinase inhibitor, clade B, member 1a | 1.93 (0.152) | 1.85 (0.059)^3^ | 2.08 (0.158)^2^ | 2.00 (0.121) |
| 67 | Stathmin | 0.87 (0.149)^4^ | 0.88 (0.094)^4^ | 0.89 (0.155)^4^ | 1.14 (0.056)^1,2,3^ |
| 68 | Superoxide dismutase 1, soluble | 2.09 (0.313) | 2.72 (0.515)^4^ | 2.25 (0.376) | 1.81 (0.248)^2^ |
| 69 | Tropomodulin 3 | 3.10 (0.274) | 3.24 (0.258) | 3.47 (0.392) | 3.45 (0.589) |
| 70 | Tropomyosin 3, gamma | 1.05 (0.142) | 0.97 (0.140) | 1.03 (0.090) | 1.13 (0.074) |
| 71 | Tubulin, beta 5 | 2.97 (0.155) | 3.13 (0.125)^3^ | 2.90 (0.091)^2^ | 3.01 (0.134) |
| 72 | Tyrosine 3/tryptophan 5 -monooxygenase activation protein, ε | 1.98 (0.131) | 2.02 (0.262) | 1.83 (0.260) | 1.93 (0.214) |
| 73 | Tyrosine 3-monooxygenase/tryptophan 5-monooxygenase activation protein, β | 2.68 (0.306) | 2.80 (0.382) | 2.52 (0.148) | 2.38 (0.194) |
| 74 | Vacuolar adenosine triphosphatase subunit B | 4.45 (0.702) | 4.33 (0.308)^3^ | 3.72 (0.377)^2^ | 3.94 (0.272) |
| 75 | Valosin-containing protein | 3.18 (0.232)^3,4^ | 2.94 (0.390) | 2.66 (0.221)^1^ | 2.61 (0.162)^1^ |
| 76 | Vimentin | 4.24 (0.102)^4^ | 4.28 (0.257) | 4.10 (0.083) | 3.97 (0.160)^1^ |

Values are means ± SD of the normalized volumes for all proteins identified by 2D-DIGE. Significance determined by t-test (p<0.05) for each group (n=4/group). Significantly different from knockout (1), KO 6hr SP-A (2), KO 18hr SP-A (3) and wild-type (4). For proteins with multiple isoforms, the normalized volumes for all isoforms were added together.
